# Integrated metabolomics and gut microbiota to reveal the anti-tumor mechanism of Jinfu’an decoction in tumor-bearing mice

**DOI:** 10.3389/fmicb.2025.1643268

**Published:** 2026-01-06

**Authors:** Peiqin Li, Huiting Peng, Zhongming Huang, Zhe Sun, Yantao Li, Siqi Wu, Lin Lin, Shuqiao Zhang, Hongyu Li, Yang Cao

**Affiliations:** 1 Shunde Hospital of Guangzhou University of Chinese Medicine, Foshan, China; 2 The First Affiliated Hospital of Guangzhou University of Chinese Medicine, Guangzhou, China; 3 The First Clinical Medical College, Guangzhou University of Chinese Medicine, Guangzhou, China; 4 Cancer Hospital of Shantou University Medical College, Shantou, China; 5 Longyan Traditional Chinese Medicine Hospital, Longyan, China

**Keywords:** Jinfu’an decoction, metabolomics, gut microbiome, lung cancer, succinic acid

## Abstract

**Introduction:**

Jinfu’an Decoction (JFAD), a traditional Chinese medicine, is used to treat lung cancer and has shown significant anti-tumor effects in clinical and experimental studies. This study integrates metabolomics and gut microbiota analysis to elucidate JFAD’s anti-tumor mechanisms.

**Methods:**

A suspension of A549-luc cells, approximately 1 × 10^6^ in number, was injected subcutaneously into the right axilla of mice to establish a tumor-bearing nude mouse model. Mice were randomly assigned to four groups: model group (MG), low-dose JFAD (JFAD-L), medium-dose JFAD (JFAD-M), and high-dose JFAD (JFAD-H), receiving treatments via gavage for 21 days. Additionally, three nude mice formed the normal group (NG), receiving no treatment. Changes in gut microbiota and serum metabolites were assessed using 16S rRNA gene sequencing and UHPLC-QE-MS non-targeted metabolomics.

**Results:**

JFAD may help restore the balance of intestinal flora in mice with lung cancer to a more normalized state. Our findings indicate that JFAD increases the abundance of Bacteroidia and decreases the presence of Firmicutes and Clostridia, thereby altering intestinal bacterial composition. Primary metabolic pathways associated with significant differences include nicotinate and nicotinamide metabolism, glycine, serine and threonine metabolism, and pyrimidine metabolism. A key differential metabolite identified was succinic acid, part of the central carbon metabolism pathway in cancer. Succinic acid showed a negative correlation with gut microbiota families Tannerellaceae and Campylobacterota. In the MG group, essential amino acid levels were markedly diminished but were significantly elevated after JFAD-M intervention. KEGG pathway analysis identified these amino acids as being linked to the PI3K/AKT and mTOR signaling pathways.

**Discussion:**

JFAD regulates the homeostasis of intestinal flora and influences amino acid and succinic acid metabolism through various pathways. These mechanisms could serve as potential targets for JFAD in inhibiting lung cancer invasion and metastasis.

## 1 Introduction

Lung cancer is the most prevalent form of cancer and the leading cause of cancer-related deaths (Freddie et al., 2024). Non-small cell lung cancer (NSCLC) is the most common type of lung cancer. However, current treatments—including surgery, chemotherapy, radiotherapy, targeted therapy, and immunotherapy—require improvement to reduce adverse reactions and inhibit tumor growth (Lester-Coll and Park, 2024; Su et al., 2025). Consequently, there is a pressing need for improved NSCLC diagnostic and therapeutic modalities.

The gut microbiota plays a crucial role in host health throughout the lifespan (Thaiss et al., 2016; Strasser and Ticinesi, 2023), and its involvement in cancer development and progression has become increasingly evident. Significant compositional differences exist between the gut microbiota of lung cancer patients and healthy individuals, with cancer patients showing reduced microbial abundance and diversity, particularly in the phylum Firmicutes and its ratio to Bacteroidetes (Liu et al., 2019). Mechanistically, the gut microbiota contributes to carcinogenesis through pathways such as immune evasion, chronic inflammation, and modulation of carcinogenic signaling (Wong and Yu, 2023). Additionally, the gut microbiota’s metabolism may contribute to chemically induced carcinogenesis (Blanka et al., 2024). Following chemoradiotherapy, changes in gut microbiota composition and function correlate with progression-free survival (PFS) in NSCLC patients (Qiu et al., 2023). Clinically, the gut microbiota serves as a valuable biomarker, capable of predicting responses to PD-1-based immunotherapy (Huang et al., 2022; Derosa et al., 2024). Consequently, these microbes are promising targets for cancer prevention and treatment.

The microbial metabolome presents significant potential for lung cancer therapy (Li et al., 2024), and serum metabolite analysis is valuable for assessing drug resistance, progression-free survival, and tumor progression in patients (Liu et al., 2024). Metabolomics, involving the qualitative and quantitative analysis of all small molecule metabolites, directly reveals the physiological and pathological state of an organism and its dynamic responses at specific times (Fiehn, 2002). Metabolic reprogramming in tumor cells is a core characteristic closely linked to drug efficacy and resistance (Hanahan and Weinberg, 2011). Metabolomics precisely captures changes in metabolic pathways related to drug resistance, progression-free survival, and tumor progression, such as altered glycolysis, amino acid metabolism, and lipid synthesis. These metabolic fingerprints serve as potential biomarkers for predicting efficacy and prognosis and help uncover new drug targets (Schmidt et al., 2021). Unlike single biomarker detection, such as PD-L1, metabolomics provides a high-dimensional, dynamic global view, offering complementary value in mechanism exploration and clinical translation (Zheng et al., 2024). The gut microbiota and serum metabolites exhibit complex interactions, serving as potential diagnostic biomarkers and therapeutic targets for lung cancer (Zhao et al., 2021; Chen et al., 2022). Specifically, intestinal microbial metabolites correlate with lung cancer patients’ responses to radiotherapy and chemotherapy (Qiu et al., 2023; Xi et al., 2022), and emaciated cancer patients display distinct gut microbiota compositions and short-chain fatty acid levels compared to healthy individuals (Ubachs et al., 2021). Therefore, analyzing gut microbiota and the serum metabolome can clarify microbial changes and identify differential metabolites between lung cancer-affected and healthy mice.

Traditional Chinese medicine (TCM) significantly contributes to lung cancer prevention and management, attributed to its synergistic effects and ability to mitigate side effects (Li Z. et al., 2021; Wei Z. et al., 2023). Jinfu’an Decoction (JFAD), developed by Professor Tietao Deng, is based on the theory of phlegm and blood stasis in lung cancer and refined through years of clinical experience. In TCM, phlegm and blood stasis is often correlated with specific pathological states observed in modern medicine, such as chronic inflammation, tissue fibrosis, disturbances in microcirculation, and metabolic dysregulation (Sun et al., 2021; Shuai et al., 2024). The effectiveness of JFAD has been confirmed in clinical practice and basic research (Sun et al., 2018; Jianning et al., 2022; Tang et al., 2023). JFAD inhibits lung cancer invasion and metastasis by regulating Lumican, p120-catenin (p120ctn), and the PI3K/Akt signaling pathway (Peng et al., 2024). Although JFAD is known to act via the PI3K/Akt pathway, the upstream regulators, particularly those involving the gut-metabolite axis, remain unidentified. This study addresses the gap by investigating whether JFAD’s anti-tumor mechanisms include a co-variation network between the microbial community and metabolites associated with disease states.

In this study, we used 16S rRNA sequencing and non-targeted metabolomics with UHPLC-QE-MS to assess the impact of JFAD on gut microbiota and serum metabolites in mice with lung cancer. The gut microbiome analysis evaluated the effects of JFAD treatment on intestinal flora. By integrating non-targeted metabolite analysis, we aimed to identify biomarkers and metabolic pathways associated with JFAD’s intervention in lung cancer, laying the groundwork for further exploration of its underlying mechanisms.

## 2 Materials and methods

### 2.1 Preparation of the JFAD

Herbs of JFAD (*Coicis Semen, Salviae Miltiorrhizae Radix et Rhizoma, Pinelliae Rhizoma, Fritillariae Thunbergii Bulbus, Persicae Semen, Cremastrae Pseudobulbus Pleiones Pseudobulbus, Phragmitis Rhizoma, Gecko, Pseudostellariae Radix, Arisaematis Rhizoma*.) were tested and approved by the First Affiliated Hospital of Guangzhou University of Chinese Medicine.

The herbs were soaked in water for 30 min before starting the decoction process. Initially, *Arisaematis Rhizoma* and *Pinelliae Rhizoma* were boiled for 1 h before adding the other herbs. The mixture was then strained and boiled again for an additional hour. The obtained extracts were subsequently filtered and combined. The herbal blend was concentrated to 11.0 g/mL. This decoction process was conducted by a qualified technician at the traditional Chinese medicine pharmacy of the First Affiliated Hospital of Guangzhou University of Chinese Medicine.

For a 70 kg adult, the recommended clinical daily dosage of JFAD is 2.36 g/kg, totaling 165 g. In an initial experiment, JFAD was mixed with double-distilled water to achieve concentrations of 1.375 g/mL, 5.5 g/mL, and 11.0 g/mL of the crude drug (Peng et al., 2024). These concentrations correspond to one times (low dose), four times (medium dose), and eight times (high dose) the adult dosage, and served as the daily dosages for mice. The resulting extracts were collected in centrifuge tubes and stored at −20 °C for future use. Research confirmed that the TCM decoction remained stable and maintained its quality under these conditions throughout the study (Yu et al., 2022; Liang et al., 2023). The decoction was analyzed using HPLC fingerprinting (Peng et al., 2024).

### 2.2 Animals and experimental design

SPF-grade BALB/c nude mice (5 weeks, 15–17 g) were utilized for the study. The experimental animals were obtained from Zhejiang Viton Lihua Laboratory Animal Technology Co., Ltd., under License No. SCXK (ZHE) 2019-0001. The NSCLC cell line A549 was from the Cell Bank of the Chinese Academy of Sciences (Beijing, China), the NSCLC cell line A549-luc was from the OBiO Technology Company (Shanghai, China).

A549-luc cell suspension (approximately 1 × 10^6^ tumor cells) was prepared by mixing with Corning Matrigel (354248) in a 1:1 volume ratio, resulting in a protein concentration of 18 to 22 mg/mL. The suspensions were stored at 4 °C. Twenty-four BALB/c nude mice were randomly selected, and 0.2 mL of the cell suspension (approximately 1 × 10^6^ cells) was injected into the subcutaneous tissue of the right axilla following sterilization. Twenty-four hours post-inoculation, the tumor-bearing mice were randomly divided into four groups of six: the untreated group, low-dose JFAD group (JFAD-L), medium-dose JFAD group (JFAD-M), and high-dose JFAD group (JFAD-H). Additionally, three mice not inoculated with tumor cells comprised the normal group (NG). JFAD was administered daily via gavage at a volume of 0.3 mL for 21 days, at the same time each day. Mice in the untreated group received an equivalent volume of sodium chloride solution in the same manner. Group details are provided in Table 1.

**Table 1:** Group settings and intervention conditions.

Group	Nomenclature	Tumor-bearing?	Intervention conditions
Negative control group	NG	No	Sodium chloride solution
Tumor-bearing model group	MG	Yes	Sodium chloride solution
Low-dose JFAD group	JFAD-L	Yes	Low-dose JFAD
Medium-dose JFAD group	JFAD-M	Yes	Medium-dose JFAD
High-dose JFAD group	JFAD-H	Yes	High-dose JFAD

At the conclusion of the experiment, the mice were euthanized, and blood and stool samples were collected for analysis. Approximately 1 mL of whole blood was obtained from each nude mouse. Blood samples were equilibrated at room temperature for 2 h. After centrifugation, the serum was collected, filtered, and stored at −80 °C. Fecal samples were collected and stored at −80 °C.

### 2.3 16S rRNA sequencing of fecal bacterial

Bacterial analysis was performed on fecal specimens stored at −80 °C. Genomic DNA was extracted using the SDS method, assessed for concentration and purity via 1% agarose gel, then diluted to 1 ng/μL. The 16S rRNA V4 regions were amplified with barcoded primers using reactions containing Phusion® Master Mix (15 μL), 0.2 μM primers, and 10 ng DNA. The primers 515F (5′-GTGCCAGCMGCCGCGGTAA-3′) and 806R (5′-GGACTACHVGGGTWTCTAAT-3′) were used (Walters et al., 2016; Snijders et al., 2016). PCR conditions included an initial denaturation at 98 °C for 1 min, followed by 30 cycles at 98 °C for 10 s, 50 °C for 30 s, and 72 °C for 30 s, and a final extension at 72 °C for 5 min. PCR products were visualized on 2% agarose gels stained with SYBR Green, pooled equally, and purified using a Qiagen Gel Extraction Kit. Libraries were constructed using the NEBNext Ultra DNA Library Prep Kit, quality-verified by an Agilent Bioanalyzer, and sequenced on an Illumina NovaSeq platform with 250 bp paired-end reads.

Barcodes are used to separate and obtain the original data for each sample, followed by the removal of barcodes and primers. R1 and R2 sequence data are concatenated using FLASH software. Quality control is performed on concatenated tags to obtain clean tags, followed by chimeric filtering to yield effective tags for analysis. To ensure accurate and reliable outcomes, raw data undergo concatenation and filtering to produce clean data. Based on clean data, denoising is performed using DADA2 (Callahan et al., 2016), and sequences with an abundance of less than five are filtered out to obtain the final ASVs. Species annotation for each ASV is conducted using the classify-sklearn algorithm in QIIME2 (Bokulich et al., 2018; Bolyen et al., 2019) with a pre-trained Naive Bayes classifier (SILVA 138.1).

To analyze the diversity, richness, and uniformity of the communities in the sample, alpha diversity was calculated using three indices in QIIME2: Chao1, Shannon, and Simpson. Beta diversity, based on weighted UniFrac distances in QIIME2, was calculated to evaluate the complexity of community composition and compare differences between groups. Two-dimensional PCoA results were visualized using the ade4 and ggplot2 packages in R software (Version 2.15.3). MetaStat and *T*-test analyses were performed using R software (Version 3.5.3) to identify significantly different species at each taxonomic level (Phylum, Class). LEfSe software (Version 1.0) was employed to perform LEfSe analysis (LDA score threshold: 4) for identifying biomarkers. The functional differences among different groups were tested using the *T*-test method.

### 2.4 Metabolomics study on serum

A 100 μL aliquot of each sample was transferred to a sterile and enzyme-free EP tube, with 400 μL of extraction solution (acetonitrile:methanol = 1:1, containing isotopically labeled internal standards) added. The mixture was vortexed for 30 s, sonicated in an ice-water bath for 10 min, and incubated at −40 °C for 1 h to precipitate proteins. Samples were centrifuged at 12,000 rpm (RCF = 13,800 × g, *R* = 8.6 cm) at 4 °C for 15 min, and the supernatant was transferred to a fresh glass vial for analysis.

LC–MS/MS analyses were performed using a Thermo Fisher Scientific Vanquish UHPLC system equipped with a UPLC BEH Amide column (2.1 mm × 100 mm, 1.7 μm), coupled to a Q Exactive HFX Orbitrap mass spectrometer. The mobile phase included 25 mmol/L ammonium acetate and 25 mmol/L ammonia hydroxide in water (pH = 9.75) as phase A and acetonitrile as phase B. The autosampler temperature was kept at 4 °C, with an injection volume of 2 μL. The mass spectrometer operated in information-dependent acquisition (IDA) mode, controlled by Xcalibur software, continuously evaluating full-scan MS spectra. ESI source parameters included: sheath gas flow rate at 30 Arb, auxiliary gas flow rate at 25 Arb, capillary temperature at 350 °C, full MS resolution of 60,000, MS/MS resolution of 7,500, collision energy of 10/30/60 in NCE mode, and spray voltage of 3.6 kV (positive ion mode) or −3.2 kV (negative ion mode). Raw data were converted to mzXML format using ProteoWizard and processed with an in-house program developed in R and based on XCMS for peak detection, extraction, alignment, and integration. Metabolite annotation was conducted using an in-house MS2 database, BiotreeDB version 2.1, which is proprietary and not publicly accessible. This comprehensive database consists of a self-constructed library containing 3,000 standard compounds, an integrated public library with 200,000 substances, and a lipid database comprising 190,000 entries. The confidence level of metabolite identification was determined based on score values from the mean table, using a cutoff threshold of 0.3.

The data were subjected to logarithmic (LOG) transformation and UV scaling using SIMCA software (version 16.0.2, Sartorius Stedim Data Analytics AB, Umea, Sweden). OPLS-DA modeling analysis was conducted on the first principal component. A permutation test was performed by randomly rearranging the order of the categorical variable Y multiple times to obtain different random Q values, validating the model’s validity.

Screening for differential metabolites primarily involves three parameters: VIP, FC, and *p*-value. VIP (Variable Importance in Projection) indicates the importance of a variable in the projection of the first principal component of the PLS-DA model, showing each metabolite’s contribution to group differentiation. FC (Fold Change) is the ratio of the average quantitative values of each metabolite in the comparison group. Significance thresholds are set at VIP > 1.0 and *p*-value < 0.05.

KEGG analysis: Pathway enrichment analysis was conducted using the Kyoto Encyclopedia of Genes and Genomes (KEGG) Pathway database^1^ to interpret the biological functions of identified differential metabolites. All pathways corresponding to differential metabolites in *Mus musculus* (mouse) were identified, and these metabolites were marked on the KEGG pathway map.

K-Means analysis: To study trends in metabolite content changes across different groups, the relative content of all differentially expressed metabolites was z-score standardized according to the screening criteria across all group comparisons, followed by K-Means clustering analysis.

### 2.5 Correlation analysis between gut microbiota and serum metabolites

Spearman correlation analysis calculated the correlation coefficient between changes in gut microbiota and metabolites. Inter-group differences in intestinal microbiota were identified based on LEfSe analysis results. Differential metabolites were identified by combining the metabolomic analysis results of JFAD, which can regulate the health of mice, with those of lung cancer-bearing mice. Spearman correlation analysis evaluated potential associations between inter-group differences in intestinal microbiota and differential metabolites, assessing the existence and strength of the correlations. This correlation is considered the most likely target for JFAD’s anti-tumor effect. Spearman’s correlation coefficient, ranging from −1 to 1, described the relationship between these parameters.

We incorporated a supervised multi-omic factor analysis using the DIABLO implementation of block sPLS-DA from the mixOmics package. This model jointly extracts latent components from both the microbiome and metabolome datasets, allowing us to identify features that co-vary across omics layers and contribute directly to the separation of the NG, MG, JFAD-L, JFAD-M, and JFAD-H groups. To further illustrate cross-omic interactions, we added a DIABLO-based Circos plot, which visualizes all significant correlations between the selected microbial features and metabolites identified by the factor model. This provides an intuitive global view of microbe-metabolite connectivity and highlights several key cross-omic pairs that appear to be modulated by JFAD treatment. In addition, we constructed Clustered Image Maps (CIM) based on DIABLO latent components to map block-to-block correlation structures. We also complemented the factor-based analysis with a high-resolution correlation matrix focusing on the top 30 microbes and top 30 metabolites showing the strongest cross-omic associations. This targeted heatmap provides a refined view of microbe–metabolite pairs most relevant to phenotype discrimination and supports the interaction patterns identified by DIABLO. Additionally, to deepen the mechanistic interpretation, we built an integrated correlation network summarizing all high-confidence microbe-metabolite associations. This network highlights several taxa and metabolites that function as central nodes, providing further evidence for coordinated functional pathways potentially involved in the therapeutic effects of JFAD.

### 2.6 Statistical analysis

Significance levels were determined using appropriate statistical analyses according to data distribution and the number of test groups. GraphPad Prism version 10.1.2 was used for analysis if the data followed a normal distribution. Pairwise comparisons were conducted using one-way ANOVA when the variance was homogeneous.

## 3 Results

### 3.1 JFAD inhibits the proliferation of A549 cells in nude mice

**Figure 1:**
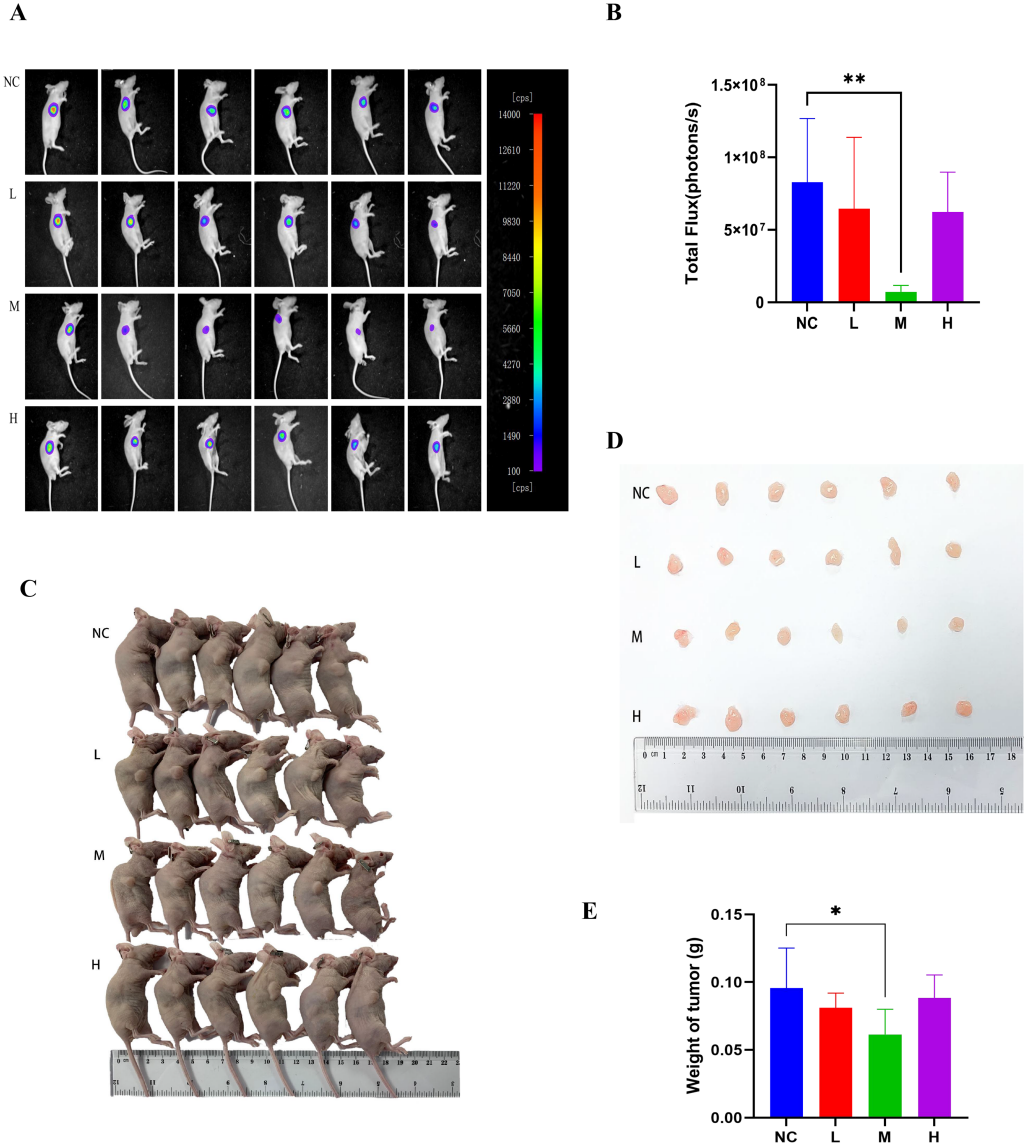
This figure is cited from our previous work (Peng et al., 2024). Anti-tumorigenic effects of JFAD in nude mice model. The A549-luc tumor-bearing nude mice were randomly divided into 4 groups [negative control group (NC), low-dose group (L), medium-dose group (M), and high-dose group (H)], gavaged with saline adjusted to the same volume of liquid for 21 days. **(A)** Representative bioluminescence imaging tracer results of nude mice at the end of study. **(B)** Quantification of bioluminescence imaging signal intensities in nude mice (*n* = 6 per group). **(C)** The transplanted tumors were harvested on day 21. **(D)** Presentation of each group of tumors. **(E)** Measurement of tumor weight. *p < 0.05, **p < 0.01 compared with NC group.

In this research (Peng et al., 2024), tumor weight decreased with JFAD treatment, with tumor inhibition rates of 15.24% (low), 35.65% (medium) and 7.57% (high). The medium dose produced the greatest and statistically significant tumor inhibition (*p* < 0.05), indicating that medium-dose JFAD effectively suppresses lung adenocarcinoma in this model. The bioluminescence imaging data was also presented. On a molecular level, JFAD was found to attenuate phosphorylation within the PI3K/Akt pathway, indicating one possible mechanism for its antitumor effects. This earlier work suggests that JFAD, particularly at medium doses, may exert antitumor activity by modulating crucial signaling pathways, warranting further exploration into its therapeutic potential (Figure 1).

### 3.2 JFAD regulates intestinal flora imbalance in mice with lung cancer

**Figure 2:**
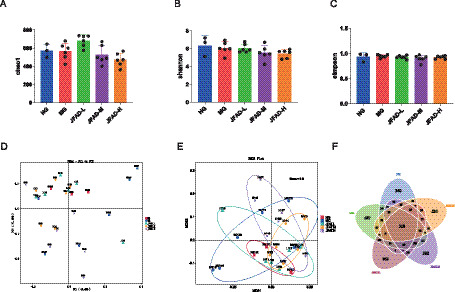
JFAD regulates gut microbial richness and diversity in mice with lung cancer. **(A)** Chao1 index. **(B)** Shannon index. **(C)** Simpson index. **(D)** PCoA analysis. **(E)** NMDS analysis. **(F)** Venn diagram of the negative control group (NG), the model group (MG), the low-dose JFAD (JFAD-L), the medium-dose JFAD (JFAD-M), and the high-dose JFAD (JFAD-H). The statistical test method used was the one-way analysis of variance.

To evaluate the regulatory effects of JFAD on the intestinal microbiome, an alpha diversity assessment was initially conducted, revealing no significant differences in the alpha diversity index between the MG group and other groups (Figures 2A–C). Subsequently, beta diversity analysis was performed to examine community structure variations, with PCoA analysis indicating that the NG group was more distant from the MG group, suggesting a more pronounced disparity in species composition. This suggests that the intestinal microbiome of tumor-bearing mice underwent notable changes. Following JFAD intervention, each group (JFAD-L, JFAD-M, JFAD-H) was positioned between the MG and NG groups, with intestinal microbiota structures closely resembling that of the NG group (Figure 2D). This implies that JFAD might assist in restoring the disrupted intestinal flora in lung cancer-afflicted mice to a healthier state. Similarly, NMDS analysis revealed that the species composition of the NG and JFAD groups was more comparable to the MG group, consistent with PCoA results (Figure 2E). Additionally, the Venn diagram shows the number of shared and unique ASVs among the groups, with 318 shared ASVs: 360 unique to the NG group, 647 to the MG group, 952 to the JFAD-L group, 392 to the JFAD-M group, and 531 to the JFAD-H group (Figure 2F). These results suggest that the sequencing depth for each classification unit is sufficient.

**Figure 3:**
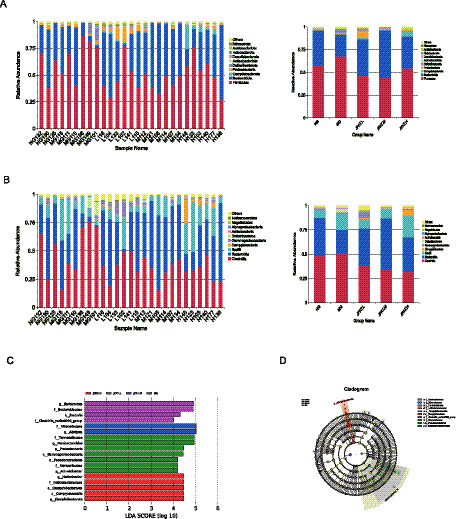
Gut microbial community structure in mice with lung cancer after JFAD treatment. **(A)** Relative abundances of top 10 at the phylum level. **(B)** Relative abundances of top 10 at the class level. **(C)** LEfSe analysis of five groups. **(D)** Cladogram of five groups.

The taxonomic composition at the phylum and class levels was analyzed. At the phylum level, Firmicutes and Bacteroidota were significantly dominant across all groups (Figure 3A). Compared to the NG group, the MG group showed a higher abundance of Firmicutes and a lower abundance of Bacteroidota. The JFAD intervention resulted in a reduction of Firmicutes and an increase in Bacteroidota. The Firmicutes to Bacteroidota (F/B) ratio was 1.732 (SD: 0.9933) in the NG group, 10.73 (SD: 13.85) in the MG group, 5.78 (SD: 11.84) in the JFAD-L group, 0.8822 (SD: 0.2861) in the JFAD-M group, and 2.23 (SD: 1.599) in the JFAD-H group. At the class level, Clostridia and Bacteroidia were clearly dominant across all groups (Figure 3B). Compared to the NG group, the MG group showed a higher abundance of Clostridia and a lower abundance of Bacteroidia. The JFAD intervention resulted in a reduction of Clostridia and an increase in Bacteroidia. Therefore, JFAD may enhance the abundance of Bacteroidia while reducing Firmicutes and Clostridia, thus altering the composition of intestinal bacteria.

To identify key phylotypes and biomarkers of gut microbiota across different groups, a comparative analysis using LEfSe was performed. This analysis revealed significant differences in bacterial community dominance between groups. In the LEfSe analysis (Figures 3C,D), the bar chart length represents the LDA score, indicating the extent to which the different species affect group differences. LDA scores greater than 4 were considered statistically significant. Bacteroidaceae and Clostridia exhibited higher LDA scores, suggesting these bacterial taxa can serve as biomarkers, with notable differences observed between groups.

**Figure 4:**
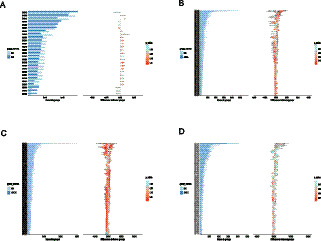
The functional differences among different groups were tested using the *T*-test method. **(A)** NG vs. MG; **(B)** JFAD-L vs. MG; **(C)** JFAD-M vs. MG; **(D)** JFAD-H vs. MG.

By comparing the MG with NG group, we revealed the significant impact of herbal JFAD on the metabolic functions of microbial communities. Compared to the NG group, the MG group showed abnormal expression in multiple metabolic pathways (Figure 4A). Specifically, the arginine metabolism pathway marker K07052 and the amino sugar metabolism enzyme K00604 were upregulated in the MG group, while pathways involved in core nitrogen metabolism, including K00278, K00334, K12267, K03340, and K00335, were downregulated. Following JFAD-L intervention, the elevated levels of K07052 and K00604 in the MG group were suppressed. In contrast, the nitrogen metabolism module K03340 and phosphonate metabolism-related markers K01546, K01547, and K01548 were significantly upregulated (Figure 4B). JFAD-M intervention further enhanced nitrogen and phosphonate metabolism functions, including the upregulation of K00278, K00334, and K12267, as well as stress-adaptive transport and regulatory factors K18331 and K18332. This suggests that medium-dose intervention may have a more profound impact on improving microbiota function (Figure 4C). JFAD-H, in the above metabolic pathways, showed no significant differences in regulation (Figure 4D).

### 3.3 JFAD changed the metabolic profile of mice with lung cancer

**Figure 5:**
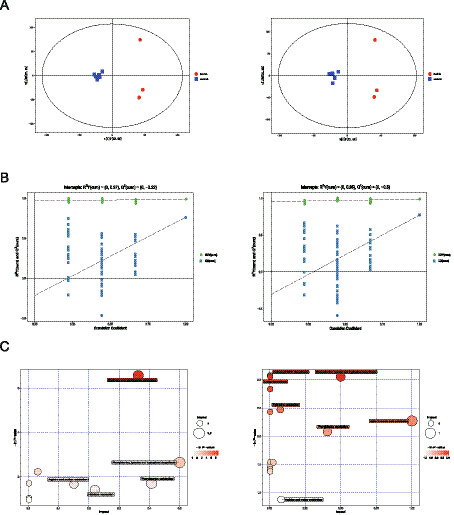
Serum metabolite profiles and key metabolic pathways regulated based on JFAD. **(A)** OPLS-DA score plot in the positive ion mode and negative ion mode. **(B)** OPLS-DA model permutation test in the positive ion mode and negative ion mode. **(C)** Metabolic pathway analysis in the positive ion mode and negative ion mode.

The OPLS-DA model was used to assess the extent of separation among various groups. The OPLS-DA score plot illustrates that the serum metabolome of the NG group is clearly distinguishable from that of the MG group (Figure 5A). This indicates that there are differences in the metabolic profiles between the two groups. The scattered points are relatively concentrated, indicating strong repeatability within each group, with similar metabolite profiles, thus demonstrating the reliability of each group’s model. Permutation test results for the OPLS-DA model, indicated by R^2^Y and Q^2^ values, suggest that the model does not suffer from overfitting, as the blue regression line crosses the left ordinate below the zero mark (Figure 5B). Furthermore, an analysis of metabolic pathways involving differing metabolites between the NG and MG groups showed that these metabolites were mainly associated with the metabolism of nicotinate and nicotinamide, alpha-Linolenic acid metabolism, as well as the synthesis of phenylalanine, tyrosine, and tryptophan (Figure 5C). The combined analysis of the two ion patterns yielded results that were nearly identical to the current results.

**Figure 6:**
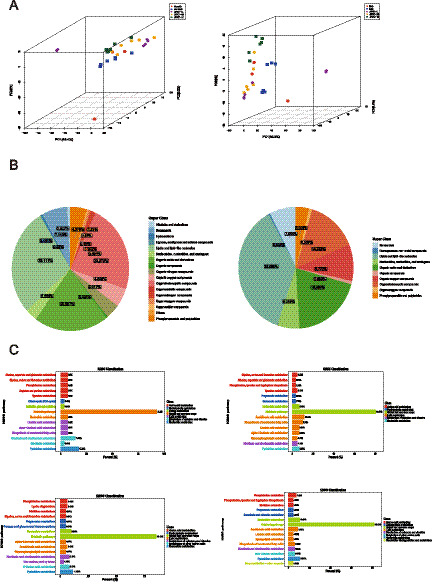
Metabolic pathway analysis of potential marker. **(A)** 3D PCA score in the positive ion mode and negative ion mode. **(B)** Pie plot of metabolite classification and proportion in the positive ion mode and negative ion mode **(C)** KEGG pathway annotation in the combined analysis of positive and negative ion modes (MG vs. NG) (JFAD-L vs. MG) (JFAD-M vs. MG) (JFAD-H vs. MG).

The first principal component (PC1) accounts for the largest variance, providing the most explanation for differences in the original data. In comparison, the second and third principal components are less significant. In positive ion mode, the scores of the first principal component are relatively similar, resulting in samples from each group clustering together. Closer sample points indicate more similar types and contents of metabolites. In negative ion mode, the detection results reveal that post-JFAD intervention, the distribution points align closely with those of the NG group, clustered around −20 on the PC1 axis. Conversely, the MG group is positioned nearer to 0 on the PC1 axis. This suggests that cancer may disrupt the metabolic pattern of mice, while JFAD intervention appears to regulate the metabolic pattern in lung cancer-bearing mice (Figure 6A).

In positive ion mode, identified metabolites were categorized by their chemical classifications, showing that a substantial portion consisted of lipids and lipid-like molecules (28.111%), organoheterocyclic compounds (23.272%), and organic acids and derivatives (20.507%) (Figure 6B). In negative ion mode, the metabolites were similarly categorized, with lipids and lipid-like molecules (36.889%), organic acids and derivatives (19.556%), and organoheterocyclic compounds (14.222%) being predominant. In both modes, the top three categories with the highest proportions of identified metabolites were lipids and lipid-like molecules, organic acids and derivatives, and organoheterocyclic compounds, with slight differences in proportions. Positive and negative ion patterns were combined for analysis. Using KEGG pathway annotation, comparisons were made between the MG group and the NG group. It was observed that metabolites related to metabolic processes such as pyrimidine metabolism (17.24%), nicotinate and nicotinamide metabolism (13.79%), glycine, serine, and threonine metabolism (6.9%), and alpha-linolenic acid metabolism (6.9%) had relatively high occurrence rates. Following JFAD intervention, the proportions of metabolites associated with these metabolic pathways decreased (Figure 6C).

Differential metabolites associated with lung cancer were identified through a screening process. Using data from the NG and MG groups, metabolites with a VIP (Variable Importance in Projection) score above 1 and a *t*-test *p*-value below 0.05 were selected. Sixty-seven differential metabolites were identified in POS mode, with significant differences noted after the JFAD intervention (Table 2). Metabolites related to nicotinate and nicotinamide metabolism included 1-methylnicotinamide, niacinamide, and N1-methyl-4-pyridone-3-carboxamide. Compared to the NG group, the relative levels of these metabolites were significantly lower in the MG group. Significant increases in the levels of 1-methylnicotinamide and niacinamide were observed in the JFAD-M and JFAD-H groups. Additionally, differential metabolites associated with glycine, serine, and threonine metabolism included betaine aldehyde and guanidoacetic acid. The levels of these metabolites were significantly higher in the MG group compared to the NG group, but JFAD treatment reduced their levels across all doses. Using the same screening technique, 34 distinct metabolites were identified in NEG mode in the NG and MG groups (Table 3). Notably, succinate, a metabolite associated with pyrimidine metabolism, increased in concentration in the MG group relative to the NG group, but JFAD treatment successfully reduced its levels. The Euclidean distance matrix for the quantitative values of differential metabolites across each group was computed, and the complete linkage approach was used to cluster these metabolites.

**Table 2:** Potential differential metabolites in serum in positive ion mode, fold change (FC) between different groups.

NO.	MS2 name	VIP	FC (MG/NG)	FC (JFAD-L/MG)	FC (JFAD-M/MG)	FC (JFAD-H/MG)
P1	Beta-Guanidinopropionic acid	1.76	1.37	1.45	0.78*	0.94
P2	1-Methylnicotinamide	1.68	0.44	1.47	2.07*	1.62*
P3	Niacinamide	1.93	0.36	1.67	1.74**	2.42**
P4	fluvoxamino acid	1.81	0.22	2.93	4.65*	2.95*
P5	3-Amino-2-piperidone	1.81	0.59	2.28**	1.18	1.35
P6	Betaine aldehyde	1.80	1.19	0.74*	0.88*	0.89**
P7	Aminofructose 6-phosphate	1.80	1.43	0.55*	0.89	0.58****
P8	3-Methylguanine	1.85	1.71	0.94	0.71**	0.67**
P9	3-Dehydroxycarnitine	1.54	1.22	0.41**	0.77***	0.82**
P10	2-Pyrrolidinone	1.93	0.65	2.23	1.38*	1.41***
P11	N-Acetyldopamine	1.85	1.79	0.62***	0.79*	0.58****
P12	N1-Methyl-4-pyridone-3-carboxamide	1.76	0.59	1.01	1.46*	1.02
P13	Norvaline	1.92	0.60	1.10	1.29*	1.08
P14	Moracin C	1.49	0.34	6.07	28.23**	24.27**
P15	N-Methylhydantoin	1.64	1.44	1.04	0.68**	0.87
P16	5-Methylcytosine	1.50	0.84	1.54	1.01	0.86*
P17	N-Nitroso-pyrrolidine	1.71	1.69	2.07	0.84**	0.78***
P18	Solasodine	1.52	1.10	390.206*	706.02****	1052.58**
P19	Prolyl-Gamma-glutamate	1.51	0.52	1.62	1.74**	2.14**
P20	4-Aminophenol	1.90	0.22	2.33***	2.83***	3.80**
P21	Leucyl-Isoleucine	1.55	0.59	0.94	1.42*	1.32
P22	o-Xylene	1.89	1.62	2.33	0.83	0.72**
P23	N-Alpha-acetyllysine	1.41	0.76	0.78	1.32*	1.07
P24	Postin	1.54	1.39	2.41	0.86	0.96
P25	3-beta-Hydroxy-4-beta-methyl-5-alpha-cholest-7-ene-4-alpha-carbaldehyde	1.93	1.72	0.47*	0.74*	0.77*
P26	L-alpha-Amino-1H-pyrrole-1-hexanoic acid	1.54	0.29	2.60	2.99**	1.79
P27	2-Methylbutyroylcarnitine	1.50	0.77	0.79	1.06	0.71***
P28	Guanidoacetic acid	1.61	1.46	0.43***	0.63**	0.62***
P29	L-2-Amino-3-methylenehexanoic acid	1.50	1.55	0.50*	0.65***	0.76*
P30	6-Methyl-3,5-heptadien-2-one	1.68	1.81	3.00	0.91	0.69*
P31	PC(18:1(11Z)/14:0)	1.48	1.82	0.79	1.05	1.68***
P32	[6]-Gingerdiol 3,5-diacetate	1.38	0.46	1.62	2.04*	1.46
P33	PC(20:5(5Z,8Z,11Z,14Z,17Z)/20:4(5Z,8Z,11Z,14Z))	1.41	1.17	0.65	0.92	0.81**
P34	[12]-Gingerol	1.79	1.99	0.20	0.63**	0.44****
P35	PC(22:6(4Z,7Z,10Z,13Z,16Z,19Z)/20:3(5Z,8Z,11Z))	1.43	1.56	0.61	0.83*	0.88
P36	Isoniazid alpha-ketoglutaric acid	1.43	1.47	0.24****	0.51****	0.55***
P37	L-Hexanoylcarnitine	1.50	1.85	0.21	0.50***	0.56**
P38	Danazol	1.78	0.34	0.65	0.53	12.56*
P39	Glaucarubin	1.61	2.00	0.44*	0.69*	0.27***
P40	4-ethylamino-6-isopropylamino-1,3,5-triazin-2-ol	1.71	1.51	0.86	0.83*	2.09****
P41	Cellulose triacetate	1.56	1.24	1.62	0.85*	0.88*
P42	Formononetin	1.77	0.26	5.69*	3.10*	3.23*
P43	Cappariloside A	1.87	0.41	1.78	1.59*	1.36
P44	Cohibin B	1.34	1.81	0.90	1.54	1.58**

The calculation of fold change (FC) utilized a binary logarithm. An FC value exceeding one signifies an increased intensity of the metabolite, whereas a value below one denotes a decreased intensity of the metabolite. *P < 0.05, **P < 0.01, ***P < 0.001, ****P < 0.0001 compared with MG group.

**Table 3:** Potential differential metabolites in serum in negative mode, fold change (FC) between different groups.

NO.	MS2 name	VIP	FC (MG/NG)	FC (JFAD-L/MG)	FC (JFAD-M/MG)	FC (JFAD-H/MG)
N1	Isopalmitic acid	1.49	1.25	0.63***	1.07	1.11
N2	Palmitoleic acid	2.02	2.40	0.24****	0.86	1.19
N3	10E,12Z-Octadecadienoic acid	1.58	1.38	0.36***	1.02	0.75*
N4	Alpha-Linolenic acid	1.70	1.71	0.34**	0.96	0.89
N5	Myristoleic acid	1.95	2.66	0.26****	0.79	1.00
N6	Ethyl dodecanoate	1.85	1.85	0.36****	1.14	1.37
N7	Thymidine	1.94	0.64	0.98	1.31*	0.97
N8	Dihydrojasmonic acid	2.01	0.27	1.37	2.21	2.80**
N9	Succinic acid	1.57	1.64	0.46*	0.71*	0.70*
N100	Hippuric acid	1.77	0.45	1.48	2.95**	3.80****
N11	L-Phenylalanine	2.03	0.52	1.35	1.54*	1.22
N12	Pseudouridine	1.66	1.88	0.50*	0.59**	0.61*
N13	Hexylresorcinol	1.61	2.31	0.25***	0.44**	0.46**
N14	12-Hydroxydodecanoic acid	1.98	3.84	0.31***	0.43***	0.56*
N15	2-Hydroxycinnamic acid	2.10	0.20	4.14	2.73*	2.68*
N16	(R)-mandelic Acid	1.87	0.55	1.18	2.19	1.54**
N17	3-Hydroxycapric acid	1.69	1.73	0.43****	0.60**	0.61**
N18	Hydroxyisocaproic acid	1.96	1.72	0.29***	0.63**	0.64**
N19	Indole-3-propionic acid	1.72	0.31	0.85	1.24	2.90**
N20	(1R,2S,3R)-2-Acetyl-4(5)-(1,2,3,4-tetrahydroxybutyl)imidazole	1.46	1.69	0.49*	0.67	0.70*
N21	Protocatechuic acid	1.61	0.15	4.25**	4.04	3.47*

The calculation of fold change (FC) utilized a binary logarithm. An FC value exceeding one signifies an increased intensity of the metabolite, whereas a value below one denotes a decreased intensity of the metabolite. *P < 0.05, **P < 0.01, ***P < 0.001, ****P < 0.0001 compared with MG group.

**Figure 7:**
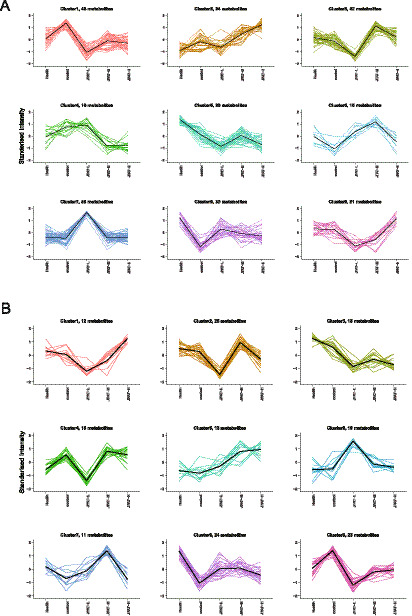
*K*-means analysis. **(A)** In the positive ion mode; **(B)** In the positive ion mode.

K-Means analysis was applied to all significant differential metabolites. In positive ion mode, a total of 301 significant differential metabolites were divided into 9 clusters, spanning from the NG group to the MG group and each JFAD group (Figure 7A). Notably, compared to the MG group, metabolite concentrations in clusters 1 and 8 showed a reversal following each JFAD intervention, suggesting these 81 metabolites may relate to the anti-tumor effect mediated by JFAD. In negative ion mode, 160 significant differential metabolites were divided into 9 clusters, spanning from the NG group to the MG group and each JFAD group (Figure 7B). Compared to the MG group, metabolite concentrations in clusters 8 and 9 showed a reversal after each JFAD intervention, indicating that these 47 metabolites (clusters 8 + 9) may also relate to the anti-tumor effect mediated by JFAD. Specific data are provided in Supplementary Tables S1, S2.

**Figure 8:**
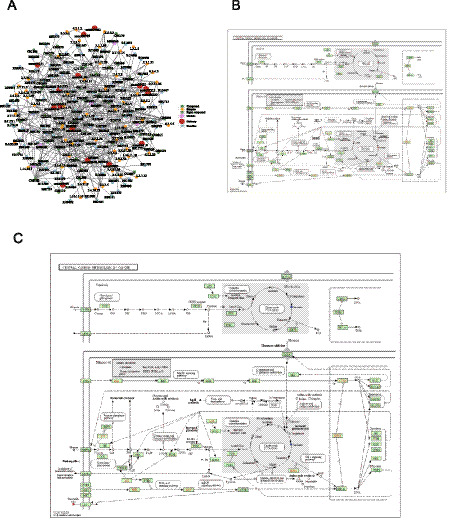
Analysis of metabolic pathways in the negative ion mode. **(A)** Network analysis (MG vs. NG). **(B)** Metabolic pathways (MG vs. NG). **(C)** Metabolic pathways (JFAD-M vs. MG). Red/blue dots representing the differentially expressed compounds.

Upon acquiring data for various groups of differentially expressed metabolites, the KEGG database specific to *Mus musculus* (mouse) was used to perform pathway searches and examine regulatory interaction networks, as shown in Figure 8A. This analysis identified 15 pathways, 5 modules, 54 enzymes, 111 reactions, and 35 compounds. It reveals intersections between the metabolic pathways of the MG and NG groups, highlighting potential targeted enzymes and metabolites. To elucidate the potential anti-tumor effects of JFAD through regulation of metabolites, the focus was directed toward the central carbon metabolism pathway (mmu05230) in cancer, as shown in the complex metabolic network in Figure 8A. The KEGG pathway diagram indicates a notable increase in succinate levels and a significant decrease in arginine levels in the MG group compared to the NG group (Figures 8B,C). Compared to the MG group, all doses of JFAD treatment (low, middle, and high) led to down-regulation of succinate. Additionally, essential amino acids were significantly down-regulated in the MG group relative to the NG group. After treatment with JFAD-L and JFAD-H, no notable changes were observed in the differential metabolites. A significant increase in essential amino acids was observed in the JFAD-M group. This observation may be associated with the PI3K/Akt and mTOR signaling pathways.

### 3.4 Correlation analysis between gut microbiota and serum metabolites

**Figure 9:**
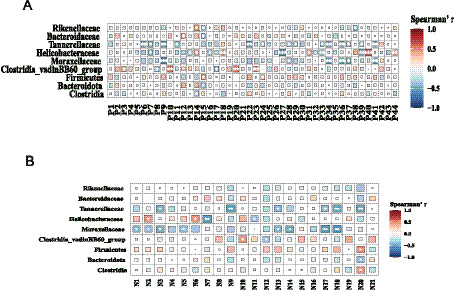
The relationship between gut microbiota and differential metabolites. **(A)** Correlation analysis in positive ion mode. **(B)** Correlation analysis in negative ion mode. Red represent positive correlations, while blue indicates negative correlations. The larger the absolute value of the correlation, the bigger the square.

Spearman correlation analysis was performed to explore relationships between six significantly different families and three differentially altered phyla with differential metabolites in both positive and negative ion modes (Figure 9). Differential metabolites in positive and negative ion modes are shown in Tables 2, 3. The results showed that 33 differential metabolites exhibited a strong correlation with these nine bacterial groups in positive ion mode. In negative ion mode, 15 differential metabolites exhibited a strong correlation with the same nine bacterial groups. These findings suggest that *in vivo* metabolite levels can reflect the gut microbiota structure. Additionally, betaine aldehyde and guanidinoacetic acid, related to glycine, serine, and threonine metabolism pathways, were negatively correlated with Tannerellaceae and Campylobacterota. Succinic acid, a differential metabolite in the central carbon metabolism pathway of cancer, was also negatively correlated with the gut microbiota families Tannerellaceae and Campylobacterota.

**Figure 10:**
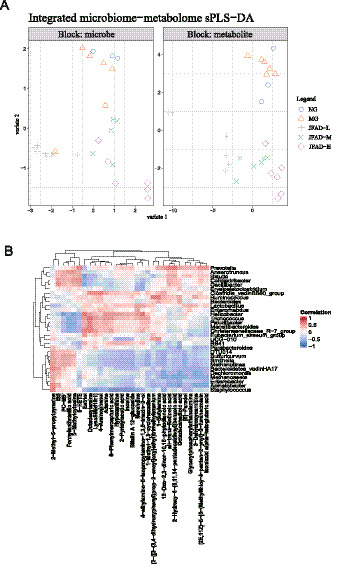
Integrated analysis of gut microbiota and serum metabolite profiles **(A)** integrated sPLS-DA analysis. **(B)** The microbe-metabolite clustered heatmap.

To further clarify the coordinated changes between gut microbiota and serum metabolites, we performed an integrated sPLS-DA analysis. The score plots showed a clear separation of the NG, MG, and JFAD groups in both the microbe and metabolite blocks (Figure 10A). MG samples clustered apart from NG, indicating a marked disruption of microbe-metabolite structure in the disease state. JFAD-treated mice shifted closer to NG along the first component, suggesting a partial restoration of the joint microbial-metabolic profile after treatment. The microbe-metabolite clustered heatmap revealed several consistent correlation patterns (Figure 10B). Taxa such as Prevotella, Anaerotruncus, Blautia, and Oscillibacter showed strong positive associations with metabolites including 2-methyl-5-propylpyrazine, and formyl-methionyl-serine. In contrast, Methanosaeta, Methanomassiliicoccus, and Staphylococcus were mainly associated with lower levels of these metabolites. These opposing clusters matched the differences observed between NG and MG mice and aligned with the reversal trend seen in the JFAD groups.

**Figure 11:**
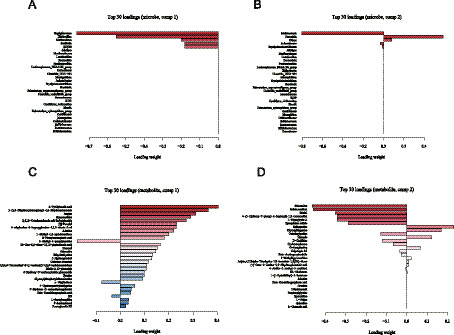
sPLS-DA components for microbiome and metabolome blocks **(A)** The load plot of the top 30 microbes ranked by contribution in the Comp1, **(B)** in the Comp2. **(C)** The load plot of the top 30 metabolites ranked by contribution in the Comp1, **(D)** in the Comp2.

The loading plots further identified variables contributing most to the group separation (Figures 11A–D). Staphylococcus, Thalassospira, Methanolinea, and IS1 showed the highest loading weights in the microbe block on component 1, whereas 2-pyridylacetic acid, 3-methyl-L-tyrosine, inosine, and bilobalide were the main contributors in the metabolite block. Component 2 highlighted additional taxa and metabolites that distinguished the different JFAD doses. These findings indicate that specific combinations of bacteria and metabolites, rather than isolated changes, are responsible for the metabolic imbalance in MG mice and the modulatory effects of JFAD.

**Figure 12:**
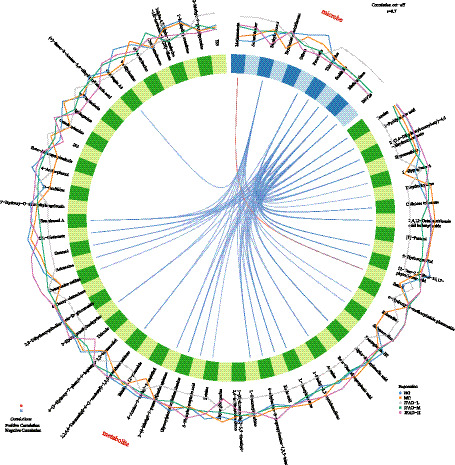
The strong microbe–metabolite associations.

The circos plot revealed a dense network of strong correlations (|*r*| ≥ 0.7) between gut microbes and serum metabolites. Although most connections were negative, these relationships still represented close associations between specific bacterial taxa and metabolite profiles. A large cluster of strong negative correlations was observed linking taxa such as Methanobrevibacter, Acinetobacter, Staphylococcus, Erysipelatoclostridiaceae, Prevotella, and Trichococcus with multiple differential metabolites involved in amino acid metabolism, steroid derivatives, and lipid-related pathways. These patterns indicate that changes in microbial composition were tightly mirrored by coordinated shifts in metabolite abundance across groups. Positive correlations were less frequent, but one notable strong positive association was identified between Methanosaeta and estriol, suggesting a distinct microbe-metabolite pair that varied in the same direction. The circos network illustrates that gut microbiota and metabolites form a highly interconnected system, in which both positive and negative associations contribute to the integrated metabolic response underlying the group differences (Figure 12).

## 4 Discussion

Dynamic changes in gut microbiota, their metabolites, and the tumor microenvironment interact to regulate tumor progression. This research aims to explore how JFAD regulates metabolism and gut microbiota in mice with lung cancer, by employing metabolomics and microbiota analyses. Non-targeted metabolomics analysis was conducted on serum metabolites, revealing differences between lung cancer and healthy mice. Additionally, JFAD can modulate serum metabolite content. Substantial changes were noted in the relative abundance of intestinal microbes at different taxonomic levels, indicating a disturbance in gut bacterial composition in mice with lung cancer. JFAD has been demonstrated to have the capacity to induce dysregulation in intestinal microbiota and serum metabolic intermediates.

Analysis of intestinal microbiota showed that, compared to the MG group, clusters from the NG group and JFAD groups were more closely related, indicating JFAD-mediated recovery of intestinal microbiota composition in lung tumor-bearing nude mice. At the phylum level, the abundance of Bacteroides in the MG group diminished compared to the NG group. This finding is consistent with previous research comparing healthy individuals with non-small cell lung cancer patients, showing that healthy individuals have higher Bacteroides presence (Vernocchi et al., 2020). Bacteroides are crucial for maintaining homeostasis in the human body, including polysaccharide breakdown and protein production (Lapébie et al., 2019; Vera-Ponce de León et al., 2020; Putnam et al., 2022). Additionally, among the myriad factors influencing the gut microbiome, the F/B ratio has emerged as a topic of considerable interest. The F/B ratio is closely related to disease states such as obesity, hypertension, and inflammatory bowel disease (Yang et al., 2015; Magne et al., 2020; Stojanov et al., 2020; Tsai et al., 2024). We found that the F/B ratio in lung cancer tumor-bearing nude mice increased. An increased F/B ratio may relate to the disease state of lung cancer. Clostridium is an anaerobic organism, meaning its spores germinate only in oxygen-lacking environments. Typically, oxygen is abundant in tissues, so anaerobic spores can proliferate rapidly only in moderately oxygen-restricted tumor areas and surroundings (Dürre, 2014). Clostridium spores colonize tumors and proliferate by using necrotic tissue. These spores target tumors highly, enabling bacterial-mediated anti-tumor therapy (Dróżdż et al., 2020). We hypothesize that JFAD may influence lung cancer invasion by modulating the F/B ratio and contributing to intestinal microbial homeostasis. LEfSe analysis highlighted key microbial shifts JFAD treatment in lung cancer, notably enriching Alistipes, known for anti-inflammatory metabolites and enhanced immune response (Parker et al., 2020). Additionally, we noted a marked change in several taxa belonging to the genus Helicobacter. The imbalance of *Helicobacter pylori* is associated with tumors (Zhuang et al., 2019). The increase in Proteobacteria is a key biomarker of intestinal microbiota imbalance and is associated with various disease states (Shin et al., 2015).

Untargeted metabolite analysis allowed the identification of specific metabolites and provided insights into the mechanistic action of JFAD. Metabolite levels in lung cancer mice were found to be different from those in healthy mice. Pyrimidine metabolism is crucial for various cellular activities, including DNA and RNA synthesis, and the production of lipids and carbohydrates. Additionally, cancer cells exhibit increased sensitivity to chemotherapy, both *in vitro* and *in vivo*, when the pyrimidine biosynthesis pathway is pharmacologically inhibited (He et al., 2022). In the MG group, succinic acid levels were higher than those in the NG group. However, JFAD reduced this increase, and notably, succinic acid is associated with purine metabolism. The succinate dehydrogenase (SDH) mutation leads to succinate accumulation, affecting the expression of genes related to purine metabolism through epigenetic modifications, such as DNA methylation (Gill, 2012). Furthermore, activation of the succinate receptor SUCNR1 may indirectly affect the activity of purine metabolic enzymes, such as xanthine oxidase, through inflammatory signals such as IL-1β (Toma et al., 2008).

In this study, we observed significantly elevated serum succinic acid levels in a lung cancer mouse model, which were effectively reduced by JFAD treatment. This finding raises a crucial question: to what extent and by what mechanisms do changes in serum succinate contribute to JFAD’s anti-tumor effects? Extensive literature suggests that succinate is not merely an intermediate of the TCA cycle; it also functions as a pivotal signaling molecule. In tumor biology, succinate accumulation is believed to promote tumor progression through various mechanisms. For instance, it can stabilize HIF-1α by inhibiting prolyl hydroxylase, thereby activating genes related to survival and angiogenesis (Wu, 2023; Selak et al., 2005). Additionally, it can interact with its receptor SUCNR1, activating oncogenic signaling pathways such as AKT/mTOR (Eniafe and Jiang, 2021). These mechanisms align with the tumor growth observed in our model. However, it’s crucial to note that our data currently establish a correlation between JFAD treatment, reduced serum succinate levels, and tumor suppression. We have yet to demonstrate that the reduction in serum succinate directly causes JFAD’s anti-tumor effect. A key unresolved question is whether blood succinate levels accurately reflect changes in the tumor microenvironment. Additionally, the activation state of HIF-1α stabilization and SUCNR1 signaling in our tumor model needs direct examination. We hypothesize that JFAD’s anti-tumor effects may partly result from its regulation of succinate levels, affecting HIF-1α and SUCNR1 signaling pathways. This hypothesis outlines future research steps: measuring succinate in tumor tissues for correlation with serum levels, verifying HIF-1α and SUCNR1 activity in patient samples or *in vitro*, and designing experiments using SUCNR1 antagonists or HIF-1α inhibitors to determine the importance of these pathways for JFAD’s efficacy.

Interactions between intestinal microbiota and serum metabolites jointly influence tumor occurrence and development. Succinic acid, a key metabolite in the central carbon metabolism pathway of cancer, is negatively correlated with the Tannerellaceae family of intestinal microorganisms. Under conditions of metabolic abnormalities and diseases, the proportion of bacterial populations producing succinic acid increases, resulting in higher succinic acid production (Wei Y. H. et al., 2023). Research has found that large numbers of bacteria from genera such as Bacteroidaceae, Barnesiellaceae, and Tannerellaceae can prolong PFS (Grenda et al., 2022). Spearman’s correlation analysis showed that Tannerellaceae are negatively correlated with liver dysfunction (Yan et al., 2023). Some studies suggest Tannerellaceae are harmful bacteria, though there is considerable controversy regarding their significance (Wang et al., 2023; Pardiñas López et al., 2025). Tannerellaceae, Bacteroidaceae, and Prevotellaceae belong to the Bacteroidetes phylum and are key components of the intestinal microbiota. The Bacteroidaceae and Prevotellaceae families have been confirmed to relate to succinic acid production (Isar et al., 2006; Serena et al., 2018). This study explored the known association between the microbiome and metabolome and, importantly, was among the first to observe potential changes in the Tannerellaceae-succinic acid association following JFAD intervention. Therefore, we introduce a new model: in tumor conditions, the decline of bacteria such as Tannerellaceae, which possess succinic acid metabolic functions, disrupts the normal clearance mechanisms of succinic acid in the gut. Future research, such as *in vitro* cultivation to verify Tannerellaceae’s capacity for succinic acid consumption or metagenomic analysis to map complete succinic acid metabolic pathways, could empirically test this hypothesis.

In this study, JFAD was demonstrated to modulate central carbon metabolism in cancer cells. Amino acids are fundamental building blocks for protein synthesis and play crucial roles in metabolic physiology and signaling. Essential amino acids (EAAs), in particular, are vital for host health and microbial interactions. Our results showed that EAA levels were significantly downregulated in the MG group compared to the NG group, while the JFAD-M group exhibited upregulation. Notably, JFAD-M administration yielded a stronger antitumor effect than either JFAD-L or JFAD-H, corroborating earlier reports that JFAD inhibits lung cancer invasion and metastasis *in vivo* through regulation of the PI3K/AKT pathway (Peng et al., 2024). Based on KEGG analysis, we identified the involvement of the PI3K, AKT, and mTOR signaling pathways in regulating EAAs within the central carbon metabolic network of cancer cells. This aligns with established mechanisms through which EAAs promote tumor progression via the PI3K/Akt/mTOR pathway (Jewell et al., 2013; Hara et al., 1998). Conversely, EAA deprivation suppresses Akt phosphorylation through a p62-dependent mechanism (Duran et al., 2011). The PI3K/AKT pathway is a central signaling cascade regulating cell growth, survival, differentiation, and metabolism, with its hyperactivation frequently linked to tumorigenesis and invasion (Ediriweera et al., 2019; Popova and Jücker, 2021; Tian et al., 2023). For instance, in small cell lung cancer (SCLC), PI3K/AKT/mTOR activation facilitates a transition from suspension to adhesion growth, conferring chemoresistance (Li X. et al., 2021). Collectively, these findings suggest that JFAD might influence EAA synthesis potentially through interaction with the PI3K/AKT/mTOR signaling pathway, which could be associated with effects on lung cancer invasion and metastasis. This mechanistic insight aligns with observations from our previous work, which indicates that JFAD could play a role in modulating PI3K/AKT-driven oncogenic processes.

The comprehensive analysis of gut microbiota and serum metabolites provides valuable insights into the complex interactions and potential mechanisms through which JFAD exerts its modulatory effects in the context of metabolic dysfunction associated with lung cancer. Our findings underscore the pivotal role of microbial-metabolite interactions in restoring homeostasis and highlight specific taxa and metabolites as potential therapeutic targets. The sPLS-DA analysis revealed a clear separation between the NG, MG, and JFAD groups, indicating a significant disruption of the microbe-metabolite axis in disease conditions, which was partially restored following JFAD treatment. This aligns with existing literature suggesting that disruptions in gut microbiota correlate with metabolic imbalances in cancer (Flint et al., 2012; Valdes et al., 2018). Taxa such as Prevotella, Anaerotruncus, Blautia, and Oscillibacter showed strong positive associations with metabolites including 2-methyl-5-propylpyrazine and formyl-methionyl-serine. Prevotella have been previously implicated in inflammatory processes and metabolic disorders (Tett et al., 2021), while Blautia is notable for its short-chain fatty acid production, which has anti-inflammatory properties (Holmberg et al., 2024). This suggests that these taxa may play protective roles in the JFAD-mediated therapeutic effects. Conversely, Methanosaeta, Methanomassiliicoccus, and Staphylococcus were associated with lower levels of these metabolites, possibly contributing to the dysregulated state observed in MG mice. The circos plot displayed a dense web of strong correlations, revealing intricate connections between gut microbes and serum metabolites. This network underscores the systemic nature of the microbial-metabolite relationship, where negative correlations were predominant. Methanobrevibacter was linked to metabolites involved in amino acid and lipid metabolism, processes often altered in cancer (Louis et al., 2014). The notable positive correlation between Methanosaeta and estriol indicates a unique microbe-metabolite pairing that may reflect specific pathways influenced by JFAD treatment.

The normal control group (NG) in our study, with a sample size of *n* = 3, presents a limitation due to its potential impact on the variability in omics analyses. Initially, we assumed high homogeneity among healthy mice, which guided our design. Our primary focus remained on the model group (MG) and JFAD treatment groups (*n* = 6 each), prioritizing these comparisons for mechanistic insights. We acknowledge that the small NG sample size may affect the robustness of the “MG vs. NG” comparisons. This has been discussed transparently to aid in the interpretation of our findings. Future studies could benefit from a larger control sample to enhance reliability.

## 5 Conclusion

In conclusion, this study’s comprehensive analysis of fecal bacteria using 16S rRNA sequencing and serum metabolomics revealed that JFAD significantly improves the internal environment of mice with lung cancer. JFAD regulates intestinal flora homeostasis and influences the metabolism of amino acids and succinic acid through various metabolic pathways. These mechanisms could serve as potential targets for JFAD in hindering lung cancer invasion and spread. This investigation identified alterations in intestinal flora and metabolites in mice with lung cancer, providing deeper insight into the mechanisms by which JFAD may impede lung cancer invasion and metastasis.

## Data Availability

The data presented in this study have been deposited in the Figshare repository, with the DOI: 10.6084/m9.figshare.30812285. Further inquiries can be directed to the corresponding authors.

## Footnotes

1
http://www.kegg.jp/kegg/pathway.html
