# Supplementary material for: Integrated metabolomics and gut microbiota to reveal the anti-tumor mechanism of Jinfu’an decoction in tumor-bearing mice
Source: Front Microbiol. 2026 Jan 6;16:1643268. doi: 10.3389/fmicb.2025.1643268 (PMC12815767; doi:10.3389/fmicb.2025.1643268)
Supplement: Supplementary file 1 [file Table_1.pdf]

Health	control	JFAT-L	JFAT-M	JFAT-H	cluster	Metabolites
-0.652746027	-0.244806381	1.76931712	-0.393914908	-0.477849804	7	1-(1-Pyrrolidinyl)-2-butanone
-0.579259772	-0.531090003	1.755881702	-0.535985617	-0.10954631	7	Adenine
-0.87597581	0.069150953	1.64425463	-0.713390067	-0.124039706	7	Beta-Guanidinopropionic acid
-0.340149194	-0.500642956	-0.49767717	-0.446610879	1.785080198	2	Adenosine
0.760668164	-1.22094078	1.198174923	-0.035173907	-0.7027284	8	Acetamidopropanal
1.141319666	-1.37417575	-0.426369812	0.782354496	-0.1231286	8	1-Methylnicotinamide
-0.027724294	0.248797075	-1.439318583	-0.140420954	1.358666755	9	Inosine
0.493581066	1.263199204	-1.222684285	0.244381075	-0.778477059	1	1,7-Dimethylguanosine
-0.156768101	0.70768341	-1.665426673	0.757123338	0.357388026	3	L-Gulose
1.214419793	-1.33999646	-0.356270991	-0.252318735	0.734166392	8	Niacinamide
0.453919194	1.366591105	0.133239536	-0.982537539	-0.971212297	4	Cytosine
-0.358031895	-1.130092853	1.587227508	-0.237303163	0.138200402	7	Beta-Aminopropionitrile
-0.607177019	0.015834547	1.725498483	-0.494327094	-0.639828917	7	Creatinine
0.658221855	-0.305808437	1.20960883	-1.409547088	-0.152475161	7	Betaine
1.619776983	-0.320577677	-1.111069322	-0.197459853	0.009329869	5	Isoquinoline
0.30020855	-0.020171992	-0.082402051	1.294586514	-1.492221021	6	Kynurenic acid
0.199667228	1.428462603	-1.358524558	-0.272089045	0.002483773	1	2-Methylguanosine
-0.239770547	-0.086457055	-0.821521631	-0.566623809	1.714373043	9	Hypoxanthine
0.185002822	-1.101648223	1.585088548	-0.244134605	-0.424308542	7	1H-Indole-2,3-dione
0.73421006	1.136509008	-1.340065625	0.075782092	-0.606435535	1	5-Methylcytidine
-0.536980907	-0.448001498	1.78563223	-0.431498884	-0.36915094	7	Methyl 2-aminobenzoate
1.122312957	-0.1040203	-1.368093096	0.806063634	-0.456263195	5	3-Formyl-6-hydroxyindole
1.594175304	-1.002572125	-0.022458157	0.096863484	-0.666008506	8	L-Phenylalanine
0.498315768	-1.671960476	0.890705507	-0.106367007	0.389306208	8	4-Amino-2-methyl-1-naphthol
-0.072516596	-0.07091991	1.671004167	-0.887729996	-0.639837664	7	Uracil
0.62911279	0.625110236	-0.541397948	-1.511029955	0.798204877	9	Indoxyl
-0.758429554	0.897845602	-1.344506719	0.396586836	0.808503835	2	Proline betaine
0.561526663	-0.481504664	-1.496971546	0.385950164	1.030999383	3	m-Aminobenzoic acid
-0.535352115	-1.281901654	0.369603213	1.389285141	0.058365415	6	(E)-5-(3,4,5,6-Tetrahydro-3-pyridylidenemethyl)-2-
1.155084098	0.755893252	-1.378594353	-0.427696766	-0.104686231	5	Uridine
-0.648963124	-0.605867248	1.737261278	-0.437843533	-0.044587372	7	Trimethylamine N-oxide
1.238313281	0.671007908	0.054632871	-0.776081328	-1.187872732	5	1-Methylhypoxanthine
0.854829731	-1.492192692	-0.182800802	0.990114928	-0.169951165	6	fluvoxamino acid
0.831777115	0.544926844	-0.255255648	-1.63711232	0.515664009	9	Pipecolic acid
-0.501847035	1.452549931	-0.761724157	-0.815657893	0.626679153	1	N4-Acetylcytidine
-0.429920558	-0.358578792	1.784014686	-0.562394238	-0.433121098	7	3alpha,4,7,7alpha-Tetrahydro-1H-isoindole-1,3(2H)-dione
0.384758774	-0.99427186	1.54048729	-0.63419777	-0.296776434	7	3-Amino-2-piperidone
-0.572540766	-0.408790429	1.783715827	-0.373490733	-0.428893899	7	2,3-Dimethylpyrazine
0.506930158	0.933736168	-1.66153264	-0.097023951	0.317890264	1	N-Ornithyl-L-taurine
-0.058207975	1.713509688	-0.475698309	-0.852932284	-0.32667112	1	7-Methylinosine
1.723850901	-0.833416571	-0.120094944	-0.278819541	-0.491519845	8	N-methylvaline
-0.184518964	0.125878684	-1.218522076	-0.269642203	1.546804559	9	2-Pyridylacetic acid
1.189612018	-1.451699936	0.543641184	-0.407430608	0.125877342	8	Vanillylamine
1.007924851	-1.585301483	0.681814339	-0.06928186	-0.035155847	8	L-Methionine
1.209008799	0.319470703	-0.603522488	0.444225431	-1.369182445	5	Imidazoleacetic acid riboside
-0.335109229	1.396789586	-1.37279673	0.112470492	0.19864588	1	Betaine aldehyde
1.695615846	0.016473531	-0.727923369	-0.727084844	-0.257081164	5	Methylpyrazine
-0.173598375	-0.146089501	1.72190611	-0.686062138	-0.716156095	7	Sphinganine
0.923679682	1.164449073	-1.114745233	-0.679667829	-0.293715694	1	N-Acetylhistamine
1.570836447	-1.017492663	0.186158672	-0.67723663	-0.062265824	8	Arecaidine
-0.213123947	1.315930178	-1.001843346	0.728658823	-0.829621708	1	Aminofructose 6-phosphate
-0.633225283	1.41732572	0.698973379	-0.772324787	-0.710749029	4	Phenylacetylglycine
-0.01940085	1.710106198	-0.626339919	-0.773452088	-0.290913342	1	Acetone cyanohydrin
0.840339407	-1.631677326	-0.227641051	0.335594019	0.68338495	8	Beta-Carboline
1.571705001	-0.494085951	-0.915670749	0.384888444	-0.546836746	5	Riboflavin
1.630471225	-0.516356478	-0.645374749	0.289353407	-0.758093405	5	Mesalazine
1.703703875	-0.001773091	-0.752262798	-0.675527795	-0.274140191	5	Histamine
-1.146369554	1.196160994	0.796686653	-0.061255956	-0.785222138	4	Prolyl-Valine
-0.269093838	-0.39399107	1.771239548	-0.656709319	-0.451445321	7	5-Aminopentanoic acid
1.346059241	0.489632234	-0.99736576	0.129586325	-0.967912041	5	Oleamide
-1.09656062	1.211152957	0.895407853	-0.40789729	-0.602102899	4	3-Methylguanine
0.09003807	1.699909097	-0.730697264	-0.469298593	-0.58995131	1	Prolylhydroxyproline
-0.004665043	-0.499024801	1.728880216	-0.688027151	-0.53716322	7	(2R)-2-Hydroxy-2-
1.210334545	-1.143204348	0.416054988	0.443366673	-0.926551859	8	Nicotinamide N-oxide
-0.09395988	-0.175765094	1.708817639	-0.670932555	-0.768160111	7	D-Alanine
0.254770223	1.09965842	-1.63085427	0.019992093	0.256433534	1	3-Dehydroxycarnitine
-0.061967156	0.23354555	-1.182333304	1.513566862	-0.502811952	3	5-(2-Hydroxyethyl)-4-methylthiazole
0.426956902	1.419738597	-0.789315291	-1.085468803	0.028088596	1	(+)-2,3-Dihydro-3-methyl-1H-
-1.161904033	0.846775584	-1.020226327	0.683523227	0.651831549	2	5-Heneicosyl-1,3-benzenediol

1.531593285	0.357368424	-0.608266203	-0.230206586	-1.05048892	5	N-Acetylglutamine
0.499021971	-0.733947277	1.533460918	-0.555196795	-0.743338817	7	Aminoacetone
-0.697255	-0.31154079	1.768504916	-0.397018274	-0.362690851	7	Melamine
0.375285719	1.569294858	-0.781037264	-0.815800018	-0.347743294	1	Asymmetric dimethylarginine
1.671683772	-1.001342513	-0.067703049	-0.2101697	-0.39246851	8	3,3,5-triiodo-L-thyronine-beta-D-glucuronoside
0.049358082	-1.131405612	1.605663957	-0.295284306	-0.228332121	7	2-Pyrrolidinone
-0.811782523	1.557968847	-0.473035254	0.434683646	-0.707834715	1	N-Acetyldopamine
-0.303727608	0.57130946	-1.62659058	0.757304633	0.601704096	3	Carbanilide
0.148682069	-0.096801578	-1.632974189	0.629317583	0.951776115	3	LysoPE(16:0/0:0)
1.355826557	0.650114654	-0.841221924	-0.16215407	-1.002565218	5	8-HETE
1.112773804	-1.443742124	0.746418581	-0.077527241	-0.337923021	8	Daidzein 4'-O-glucuronide
0.397249227	0.898093588	-1.144221958	-1.001562133	0.850441276	9	Homoeriodictyol 4'-isobutyrate
1.535055772	0.442764511	-0.716964613	-0.367134652	-0.893721018	5	beta-Alanine
1.437819418	-0.730356363	-0.700693445	0.670507133	-0.677276743	5	N1-Methyl-4-pyridone-3-
1.430135577	-0.322307714	-1.333738938	0.265357156	-0.039446081	5	Prostaglandin H2
1.662687103	-0.834463251	-0.466717625	0.17699957	-0.538505796	8	Norvaline
-0.831951327	-0.835479518	-0.495513664	0.969372516	1.193571992	2	Estriol
1.752485235	-0.120497825	-0.673691233	-0.504149264	-0.454146913	5	4-Pyridoxic acid
-0.34021852	1.485816883	-1.231187051	0.323075705	-0.237487017	1	Octadecanamide
-0.355133306	-0.383538112	1.775382392	-0.381586716	-0.655124258	7	(+)-threo-2-Amino-3,4-dihydroxybutanoic acid
-0.772403704	-1.050832895	1.472842963	-0.009855202	0.360248839	7	L-Hypoglycin A
-0.050754305	-1.041251581	0.442618176	1.44417054	-0.79478283	6	4-Acetylimidazo[4,5-c]pyridine
-0.296257644	0.15035172	1.625761902	-0.464667071	-1.015188908	7	Phytosphingosine
-0.809394707	-1.277596328	0.803299387	0.990705253	0.292986394	6	Trimethylaminoacetone
-0.078022065	0.354444406	1.508036363	-0.860911497	-0.923547206	4	2-Methylpyrrole
-0.750299375	-0.901277807	-0.503809435	1.232740847	0.922645771	2	Moracin C
-0.964428774	0.864079131	1.089341968	-1.063497728	0.074505404	4	N-Methylhydantoin
0.227447759	-0.218971729	1.560728759	-1.126101155	-0.443103633	7	(2E)-Decenoyl-ACP
-0.30288734	1.543547162	-0.281552407	0.222766071	-1.181873486	1	Furcelleran
0.247942303	-0.461085912	1.610440497	-0.419196748	-0.978100142	7	5-Methylcytosine
-1.138964281	0.160506344	1.578774683	-0.253906449	-0.346410298	7	Dihydrothymine
-0.320957779	-1.068632543	1.632722579	0.051163318	-0.294295574	7	1,2,3,4-Tetrahydro-2-methyl-b-carboline
1.054778201	-0.499716272	0.987021805	-1.258126783	-0.283956951	8	L-Asparagine
0.918484655	1.1612749	-1.068446514	-0.227383669	-0.783929372	1	gamma-Glutamylleucine
0.731437604	0.365081389	-0.211578199	-1.645672203	0.760731409	9	Indoxyl sulfate
0.178790424	0.270711328	-1.50954618	-0.195984879	1.256029307	9	2-(3,4-Dihydroxybenzoyloxy)-4,6-dihydroxybenzoate
1.565992009	-0.1935557	0.289050038	-0.678449288	-0.983037059	8	Serotonin
-0.363055541	1.426468751	-1.276286	0.407944445	-0.195071655	1	3-Methylglutarylcarnitine
0.228308647	0.076025152	1.470903329	-1.206056395	-0.569180733	4	5-Aminopentanal
1.220296625	0.776673296	-0.989685811	-0.044912234	-0.962371875	5	Pyro-L-glutaminyl-L-glutamine
-0.7995945	-0.099109399	1.731690636	-0.365797957	-0.46718878	7	N-Nitroso-pyrrolidine
-0.466342697	-0.963724354	1.672122355	-0.001714403	-0.240340901	7	2-Methyl-5-(2-propenyl)pyrazine
-0.940283185	-0.940088329	-0.087500725	0.604341259	1.363530979	2	Solasodine
1.002118373	0.167082716	-1.654749045	0.479173347	0.006374609	3	2,8-Dihydroxyquinoline-beta-D-glucuronide
1.687561354	-0.159819257	-0.509716942	-0.09152275	-0.926502405	5	L-Tyrosine
-0.785949561	-0.814299139	-0.556813429	1.281878661	0.875183468	2	Cholesterol
0.921430754	0.556075919	-1.536657268	-0.459648569	0.518799164	9	Valyl-Lysine
-0.603267384	-1.052964507	-0.215535996	0.332095514	1.539672372	2	Homocysteine thiolactone
1.655674938	-0.995256269	0.040517973	-0.248224341	-0.452712301	8	Piperidine
0.538718076	-1.590993741	-0.151566743	0.137738589	1.06610382	8	Prolyl-Gamma-glutamate
0.622172272	-0.10437611	-1.569318468	-0.015717035	1.067239341	9	LysoPE(18:3(6Z,9Z,12Z)/0:0)
0.789922733	1.121926293	-1.132819228	0.125548374	-0.904578172	1	6-Hydroxy-5-methoxyindole glucuronide
1.223279186	-1.381485751	-0.424064926	-0.059388181	0.641659672	8	4-Aminophenol
0.853957891	-1.29847204	0.126208177	1.03039877	-0.712092798	6	5-Methyldeoxycytidine
-0.032043412	-0.364345018	1.71176704	-0.846062715	-0.469315895	7	Desloratadine
1.689198205	-0.186259875	-0.444324826	-0.108497498	-0.950116006	5	Benzoic acid
-0.247489193	-0.441997033	-1.229784691	1.400779941	0.518490975	3	Guanosine
1.361584735	-0.875339083	-1.076204379	0.449295568	0.14066316	5	Leucyl-Isoleucine
-0.934246209	-0.459083166	1.693020621	-0.200837898	-0.098853347	7	Methylimidazole acetaldehyde
-0.794824782	0.149391908	-1.244798379	0.874668757	1.015562496	2	SM(d18:1/16:0)
-0.189002135	0.665507388	-1.419300026	-0.257483452	1.200278225	9	Butyrylcarnitine
0.398490721	-0.918317645	-0.851510885	-0.130585765	1.501923573	9	Octadecylamine
1.251275198	0.673578104	-1.088893914	0.05398371	-0.889943099	5	all-trans-Retinoic acid
-0.469071358	0.653194964	1.42739514	-0.898912962	-0.712605783	4	O-Acetylserine
-0.503612081	-0.420895922	1.786647642	-0.483363797	-0.378775842	7	PA(16:1(9Z)/16:0)
1.706926972	-0.878434371	-0.041990324	-0.436246869	-0.350255409	8	Nicotinic acid mononucleotide

0.85100524	0.765303431	-1.226415392	0.550035791	-0.93992907	5	Palmitoylethanolamide
0.401367038	-0.072934539	1.397672443	-0.42125067	-1.304854272	4	Acetylhydrazine
0.362608903	1.585070166	-0.472989111	-0.887334006	-0.587355953	1	1-(beta-D-Ribofuranosyl)-1,4-dihydronicotinamide
1.077422048	0.276137427	-1.600366952	0.416045588	-0.169238111	5	Valyl-Valine
0.204220581	0.411322	-1.49464837	1.203987628	-0.324881838	3	Thiamine
0.842048162	-0.784370977	-1.178947068	1.133861685	-0.012591801	3	Montecristin
-0.756359773	-0.079611331	1.730418048	-0.330566626	-0.563880318	7	Indole
-0.677443884	-0.712755271	0.047271289	1.703231229	-0.360303364	6	Laccarin
-0.537278869	-0.427495476	1.671611291	-0.845600093	0.138763147	7	L-Glutamine
-0.687975643	-0.141800161	1.751592379	-0.378344776	-0.5434718	7	o-Xylene
1.389249192	0.634224323	-1.077169226	-0.30037086	-0.645933429	5	2-Hydroxy-6-(8,11,14-pentadecatrienyl)benzoic acid
0.969439965	-0.423299605	-1.400860848	0.953344524	-0.098624037	3	N-Alpha-acetyllysine
-1.454225885	0.512013125	-0.608821499	0.932921671	0.618112589	2	Vitamin A
-0.488947766	1.681849915	-0.950005097	-0.1337321	-0.109164952	1	PC(22:6(4Z,7Z,10Z,13Z,16Z,19Z)/22:6(4Z,7Z,10Z,13Z,16Z,19Z))
0.283362333	-0.525142963	-1.169582877	1.505692089	-0.094328582	3	Isovalerylglycine
-0.632804706	1.145679892	0.861962716	-0.140195763	-1.234642139	4	alpha-Methylstyrene
-0.58128054	-0.310651076	1.780313861	-0.47139308	-0.416989165	7	Phosphatidylserine
-0.662165051	-0.186054303	1.762479871	-0.49352942	-0.420731098	7	1,3-Diisopropylbenzene
-0.578606524	-0.143229371	-1.280499422	1.014353077	0.987982239	2	PC(16:0/16:0)
-0.394539834	-0.872570049	1.713211262	-0.067545414	-0.378555965	7	N-Acetyl-L-phenylalanine
-0.712951989	-0.861267677	1.613013101	-0.290831535	0.2520381	7	D-Ornithine
-0.684613528	-0.27562394	1.766329237	-0.475588338	-0.330503431	7	Postin
0.43379453	0.134692073	-1.603877716	-0.067641631	1.103032744	9	Bitalin A 12-glucoside
-0.653078625	1.432332039	-1.193805791	0.133743414	0.280808963	1	3-beta-Hydroxy-4-beta-methyl-5-alpha-cholest-7-ene-4-alpha-carbaldehyde
0.734255264	-1.271238896	1.215706658	-0.07807075	-0.600652276	8	N-Acetylornithine
1.137230197	-1.388877573	0.221546024	0.618453892	-0.588352539	8	L-alpha-Amino-1H-pyrrole-1-hexanoic acid
1.410933547	0.118860691	-0.769818922	0.366248313	-1.126223629	5	2-Methylbutyroylcarnitine
1.649572923	-0.447797525	0.202990425	-0.861282138	-0.543483685	8	Imidazole-4-acetaldehyde
1.478034708	0.471540837	-0.195726832	-0.785379297	-0.968469415	5	Carnosine
1.665296757	-0.277687381	-0.945545281	-0.502021566	0.059957472	5	12-Oxo-2,3-dinor-10,15-phytodienoic acid
0.059732357	1.587711347	-1.164918775	-0.208446168	-0.27407876	1	Guanidoacetic acid
0.422138507	1.381547999	-1.297915357	-0.02856693	-0.477204218	1	N6-Methyladenosine
-0.797865959	-0.977354625	-0.230489219	0.588351185	1.417358618	2	Calystegin A3
-0.104386138	-0.083402536	-0.941063698	-0.544490589	1.673342961	9	Marshdine
-1.062247627	0.303120888	-0.796061394	1.461965639	0.093222494	3	PC(24:1(15Z)/18:3(6Z,9Z,12Z))
0.311430847	0.455631178	-1.741751045	0.792880934	0.181808086	3	PC(24:1(15Z)/14:1(9Z))
-0.308631276	-0.512296176	-0.5054248	-0.456475602	1.782827855	2	3-Hydroxymethylantipyrine
1.007165239	1.100538327	-0.924926267	-0.260130516	-0.922646783	5	5,12-dihydroxy-6,8,10,14,17-eicosapentaenoic acid
1.58697455	-0.936089122	-0.265269879	0.287052178	-0.672667728	8	Erinacine P
0.706914374	-0.344128435	-0.793825758	-0.937474168	1.368513989	9	Brassicanal A
-0.360649219	1.547437457	-1.135704951	-0.329053562	0.277970276	1	L-2-Amino-3-methylenehexanoic
-0.541528475	0.147960486	-1.384715717	1.194322552	0.583961154	3	PC(18:0/15:0)
-0.676629785	-0.230638565	1.760818381	-0.316723236	-0.536826794	7	6-Methyl-3,5-heptadien-2-one
-0.057667229	1.507080766	-1.308133278	-0.116407632	-0.024872626	1	Stearidonyl carnitine
-0.558913181	-0.239457468	1.775428091	-0.508661382	-0.46839606	7	Gyromitrin
0.467265614	1.175846998	0.435260094	-0.881422632	-1.196950074	4	4-Hydroxymandelonitrile
-1.561964352	0.387517741	-0.09316485	1.180613863	0.086997598	2	DG(18:0/18:4(6Z,9Z,12Z,15Z)/0:0)
-0.060518111	0.576937365	-1.434765033	-0.309998955	1.228344733	9	Questiomycin A
0.149882661	0.857979132	-1.720475359	0.473487176	0.239126389	1	3beta,6beta-Dihydroxynortropane
0.975001839	1.032150684	-1.299783042	-0.204663548	-0.502705933	1	Taurine
-1.09743203	-0.028262826	-0.53634769	0.080693814	1.581348733	2	PC(18:1(11Z)/14:0)
1.060987876	-0.731702084	-1.319303858	0.727695768	0.262322297	3	Acrylamide
0.620618495	-0.331999848	-1.247579541	1.342136541	-0.383175647	3	2-O-(5,8,11,14,17-Eicosapentaenoyl)-1-O-hexadecylglycero-3-
-0.187170845	-0.2980835	-1.375641733	1.285593093	0.575302985	3	Persicaxanthin
0.021335438	1.524495962	-0.44889737	0.111955531	-1.208889561	1	Ephedrannin A
-0.396627086	-0.537675743	1.786131321	-0.409777895	-0.442050598	7	Histidinal
1.082755223	-1.402695027	-0.080439058	0.822067928	-0.421689066	8	[6]-Gingerdiol 3,5-diacetate
0.377605904	-0.178469593	-1.301739094	1.42399013	-0.321387348	3	PC(18:2(9Z,12Z)/P-18:1(11Z))
0.573907639	0.419406835	1.047776321	-1.416693661	-0.624397134	4	DL-Glutamate
0.736432891	-1.385119991	1.156265922	-0.445283792	-0.062295029	8	Apigenin 7-sulfate
-0.639193505	-0.332402784	1.769427712	-0.525007211	-0.272824212	7	1-Methyl-1,3-cyclohexadiene
0.762570286	0.776948919	0.648262534	-1.067450784	-1.120330955	4	PC-M5'

1.107852345	-1.507551953	0.664762261	-0.239564789	-0.025497864	8	3-Methyl sulfolene
0.511904081	0.398073894	-1.777576794	0.286578024	0.581020795	3	Stigmasterol
0.762620766	-0.232566667	-1.47459812	1.082108461	-0.137564439	3	PC(16:1(9Z)/P-18:1(11Z))
1.28973651	-1.031641048	0.765040298	-0.797580495	-0.225555265	8	Ornithine
1.131721009	-1.236904112	0.623104596	0.318488429	-0.836409922	8	3alpha,4,5,7alpha-Tetrahydro-5-hydroxy-1H-isoindole-1,3(2H)-dione
0.014776686	1.197236897	-0.054979441	-1.55166113	0.394626987	4	2,4,12-Octadecatrienoic acid isobutylamide
0.711676898	-1.006525675	1.376655036	-0.547632837	-0.534173422	7	Isonicotinic acid
0.957276117	0.652159147	-0.620957351	0.450265823	-1.438743737	5	myo-Inositol
-0.348305908	-0.375795438	1.72063953	-0.87264605	-0.123892134	7	4-Amino-2-methylenebutanoic acid
1.601593406	-0.231710222	-0.642172695	0.229458284	-0.957168773	5	Pinostilbenoside
0.737691584	-0.798129569	-1.340143419	0.845207574	0.555373829	3	PC(P-18:1(9Z)/15:0)
-0.136518837	-0.938817474	1.571874167	0.254682878	-0.751220735	7	Methylguanidine
-0.189817064	-0.752857582	-0.592105783	-0.199256103	1.734036531	2	Armillaramide
0.031090226	1.164726442	-1.497606989	0.571656566	-0.269866245	1	PC(20:5(5Z,8Z,11Z,14Z,17Z)/20:4(5Z,8Z,11Z,14Z))
-0.63505027	-0.460718337	1.769201926	-0.219204751	-0.454228568	7	Tromethamine
-0.306242376	-0.477788789	1.78252112	-0.531345157	-0.467144798	7	3-Methylcytosine
0.155190987	0.063669344	-1.488654768	1.323947289	-0.054152852	3	LysoPC(24:1(15Z))
0.450653672	-0.292509359	-1.406578564	1.314721378	-0.066287127	3	PC(20:5(5Z,8Z,11Z,14Z,17Z)/P-18:0)
-0.823472349	-0.217295111	-0.921448884	0.48087743	1.481338913	2	SM(d17:1/24:1(15Z))
-0.836648668	0.22814552	-1.240143527	0.899169851	0.949476825	2	SM(d16:1/24:1(15Z))
1.113448157	0.20278777	-0.824019069	0.733423137	-1.225639995	5	9-HODE
0.239195576	0.829045821	-1.424728622	-0.58401464	0.940501865	9	PC(22:5(7Z,10Z,13Z,16Z,19Z)/16:1(9Z))
0.030640993	-0.337250686	-1.239168912	1.532845803	0.012932801	3	PC(P-18:1(9Z)/16:0)
1.164368239	-1.351690025	0.275560326	0.563068578	-0.651307118	8	Alanyl-Lysine
0.481826435	0.008910382	-1.257195533	1.356238557	-0.58977984	3	PC(20:5(5Z,8Z,11Z,14Z,17Z)/P-18:1(11Z))
-0.87124217	-0.148780708	-0.67067962	0.026891488	1.66381101	2	N-Palmitoylsphingosine
-1.381645917	-0.101280044	-0.096427772	0.14768018	1.431673553	2	L-Cyclo(alanylglycyl)
-0.592943491	0.386534935	-1.373661263	1.216504826	0.363564992	3	PC(18:3(6Z,9Z,12Z)/18:1(11Z))
-1.083501737	0.375304759	-1.00966517	0.550530857	1.167331291	2	SM(d18:1/20:0)
-0.94611356	0.300192074	-1.116430875	0.553004711	1.209347651	2	SM(d18:1/22:0)
0.083585673	-0.253442177	-1.5459534	1.040670878	0.675139027	3	PC(18:2(9Z,12Z)/18:0)
1.139883014	0.081599777	-1.195915282	0.782033199	-0.807600709	5	N-acetyltryptophan
0.117290114	-1.617810651	0.894185464	0.742225034	-0.135889962	6	Valyl-Tyrosine
0.914804464	0.595271908	-1.09956798	0.665227233	-1.075735625	5	PC(P-18:1(11Z)/22:6(4Z,7Z,10Z,13Z,16Z,19Z))
-0.420568154	1.128798276	-1.486521335	0.523909463	0.25438175	1	2-Methyl-4-oxopentanedioic acid
-1.040733441	-0.04359269	-0.63572946	0.141861375	1.578194217	2	SM(d18:0/18:1(9Z))
-1.166401898	0.0400564	-0.765863627	0.584768627	1.307440498	2	SM(d18:1/18:1(9Z))
-0.170282375	1.518849615	-1.200414134	0.251600431	-0.399753536	1	[12]-Gingerol
-0.080244551	0.342940106	-1.352136622	1.397433419	-0.307992353	3	PC(P-18:1(11Z)/22:5(4Z,7Z,10Z,13Z,16Z))
-0.922265853	1.256736212	-1.106431943	0.231245855	0.540715729	1	PC(22:6(4Z,7Z,10Z,13Z,16Z,19Z)/20:3(5Z,8Z,11Z))
1.409371018	0.100260621	-0.193841672	0.083857983	-1.399647951	5	apo-[3-methylcrotonoyl-CoA:carbon-dioxide ligase (ADP-
-0.097225312	-0.507785443	1.727462517	-0.317683836	-0.804767926	7	3-Pyridylacetic acid
-0.480065802	-0.435467266	-0.502301795	-0.368688744	1.786523608	2	PC(20:2(11Z,14Z)/14:0)
0.584654597	1.431663709	-0.873254576	-0.261171488	-0.881892242	1	Cervonyl carnitine
0.294530172	0.082762465	-1.353489689	1.381963077	-0.405766025	3	PC(20:2(11Z,14Z)/15:0)
-1.127910495	0.550983653	-1.047723253	0.754157411	0.870492684	2	PC(22:4(7Z,10Z,13Z,16Z)/16:0)
1.460836603	-0.045088917	-1.224784129	0.320206631	-0.511170188	5	(E,E)-Boviquinone 3
-1.103418571	-0.082184987	-0.818853677	0.782834749	1.221622486	2	PI(20:1(11Z)/18:2(9Z,12Z))
1.402057597	-0.504651541	-0.413779114	0.621725947	-1.105352889	5	Diferuloylputrescine
-1.478181729	-0.235476867	0.85923269	-0.14559888	1.000024786	2	Arsenobetaine
0.072244856	-0.13067857	-1.479868044	1.318927295	0.219374464	3	PC(22:4(7Z,10Z,13Z,16Z)/P-18:0)
-0.353228951	1.692830455	-0.969596283	-0.207200309	-0.162804912	1	3-Hydroxyisovalerylcarnitine
0.306495457	1.454105065	-1.293880071	-0.298932195	-0.167788256	1	Isoniazid alpha-ketoglutaric acid
1.396393966	0.36645701	-0.995322557	-0.945269429	0.17774101	5	Glycerylphosphorylethanolamine
0.23493694	0.826939952	-1.737778863	0.417568304	0.258333667	1	LysoPC(18:2(9Z,12Z))
0.372406146	0.126882391	-1.506556525	1.234458036	-0.227190048	3	PC(16:1(9Z)/16:1(9Z))
-0.393227772	-0.48583492	1.787419171	-0.423248398	-0.485108081	7	13-L-Hydroperoxylinoleic acid
-0.712324173	-0.250621066	1.763139103	-0.42210544	-0.378088424	7	6,10,14-Trimethyl-5,9,13-pentadecatrien-2-one

0.334306595	1.380936503	-1.301445665	0.110810662	-0.524608094	1	PC(20:4(5Z,8Z,11Z,14Z)/20:4(8Z,11Z,14Z,17Z))
-1.013197613	0.007613009	-0.730882183	0.18576529	1.550701496	2	PC(22:2(13Z,16Z)/16:1(9Z))
0.990528891	-0.101012352	1.048399923	-0.842969112	-1.09494735	4	3-Methyl-alpha-ionyl acetate
-0.070738932	1.542823651	-1.249133396	-0.232932025	0.009980702	1	L-Hexanoylcarnitine
0.97982206	-0.058417469	-1.579736855	-0.073491858	0.731824122	9	(6beta,22E)-6-Hydroxystigmasta-4,22-dien-3-one
0.532012899	0.87793309	-1.217411266	0.753900494	-0.946435217	1	LysoPC(20:0/0:0)
-0.561992611	-0.373289311	1.784284842	-0.443789006	-0.405213914	7	2-Methoxy-6-methylpyrazine
-0.286119663	0.876011554	-1.261503011	-0.48083962	1.15245074	9	LysoPC(16:1(9Z)/0:0)
1.697608493	-0.105676293	-0.810568949	-0.121728435	-0.659634816	5	1-(Hydroxymethyl)-5,5-dimethyl-2,4-imidazolidinedione
-0.176981054	1.672493615	-0.62351086	0.012598937	-0.884600638	1	Biliverdin
1.151491441	0.311802953	-1.370981157	0.542484264	-0.634797502	5	Octadecanedioic acid
-0.000899496	-0.728440254	-1.182150515	1.305742724	0.605747542	3	PE(20:5(5Z,8Z,11Z,14Z,17Z)/P-18:0)
-0.121854286	-0.491939417	-0.559670156	-0.584038702	1.757502561	9	Danazol
0.330174955	1.566364716	-0.272246299	-0.960813338	-0.663480035	1	Glycylprolylhydroxyproline
-0.281420703	1.506660327	-0.488360618	0.386804041	-1.123683046	1	Glaucarubin
-0.397408159	-0.453629144	-0.676820912	-0.238823466	1.766681681	2	S-Phenylmercapturic acid
-0.739164966	-0.154802446	-0.396828371	-0.458939403	1.749735187	2	4-ethylamino-6-isopropylamino-1,3,5-triazin-2-ol
0.157942603	0.473316879	1.188974543	-0.32670513	-1.493528895	4	Asparaginyl-Hydroxyproline
-0.674908169	-0.095156143	1.747427946	-0.535157674	-0.442205959	7	Cellulose triacetate
0.181487599	1.018526069	-1.661092225	0.409624177	0.05145438	1	LysoPC(22:6(4Z,7Z,10Z,13Z,16Z,19Z))
0.172682563	-0.787423935	1.115945318	0.744186353	-1.245390299	6	Arbutin
0.244431988	1.290135503	-1.50766757	-0.047946921	0.021047001	1	5-Hydroxymaltol
-1.060604136	-0.437520038	1.634635679	-0.102487209	-0.034024297	7	2-(1-Propenyl)-delta1-piperideine
0.255693957	-1.406716379	1.387451191	-0.155627885	-0.080800884	7	Formononetin
-1.024506437	0.458473499	-1.113931283	0.629975186	1.049989035	2	PI(22:4(10Z,13Z,16Z,19Z)/16:0)
0.294229672	-0.439088449	-1.518820073	0.954114039	0.709564811	3	PE(P-18:1(9Z)/20:3(5Z,8Z,11Z))
-0.659808504	0.123239679	-1.189579835	1.430775808	0.295372852	3	lysoPC(26:1(5Z))
0.555093083	0.918362685	-1.165726876	-1.003044628	0.695315736	9	[8]-Paradol
0.59556839	-0.724380156	-1.22252722	1.272720727	0.078618259	3	PE(P-18:1(11Z)/16:0)
0.834637724	-1.199139007	0.987874308	0.277912038	-0.901285062	8	Thiomorpholine 3-carboxylate
1.58745203	-1.120266961	0.160283216	-0.278740893	-0.348727391	8	Methyl bisnorbiotinyl ketone
-0.125283585	0.23353345	-1.653766345	0.788764985	0.756751495	3	2-(4-Methyl-3-pentenyl)anthraquinone
-0.729484032	1.595374151	-0.310513986	-0.857340594	0.301964461	1	O-propanoyl-carnitine
-0.043892483	1.627803503	-0.726443446	-0.904645441	0.047177868	1	Deoxyuridine
-0.291551068	-0.235891236	-1.262938634	1.470345819	0.320035119	3	(2-{[3-(3,4-dihydroxyphenyl)prop-2-enoyl]oxy}ethyl)trimethylazanium
-0.182186567	-0.53255865	1.767878007	-0.486240512	-0.566892278	7	Formiminoglutamic acid
-0.631927159	-1.046058681	0.071470271	1.581356482	0.025159086	6	PC(18:4(6Z,9Z,12Z,15Z)/15:0)
1.50149139	-1.184801821	0.277064259	-0.08624472	-0.507509109	8	Cappariloside A
-0.600224421	-0.89963095	1.6127364	-0.390435303	0.277554274	7	Epinephrine sulfate
-0.302863998	-0.395497271	-1.309155492	1.110775739	0.896741023	3	PE(P-18:1(11Z)/22:4(7Z,10Z,13Z,16Z))
0.455462011	-0.505275494	-1.518800575	0.773799567	0.794814491	3	PE(P-18:1(9Z)/16:1(9Z))
-0.970335354	-0.993511291	-0.065162556	0.948552782	1.08045642	2	4-(3-Hydroxy-7-phenyl-6-heptenyl)-1,2-benzenediol
-0.213334853	0.451681694	-1.270444763	1.411545731	-0.37944781	3	4-Hydroxyphthalide
-1.276556899	-0.258212792	-0.493561296	0.975033751	1.053297236	2	Cohibin B
-0.159233647	-0.606612122	-1.252433336	1.035138067	0.983141038	3	(3beta,5alpha,6beta,22E,24R)-23-Methylergosta-7,22-diene-3,5,6-triol
0.089270017	-0.29299655	-1.413285488	1.3568873	0.260124721	3	PE(P-18:1(9Z)/20:5(5Z,8Z,11Z,14Z,17Z))
