# Supplementary material for: Integrated metabolomics and gut microbiota to reveal the anti-tumor mechanism of Jinfu’an decoction in tumor-bearing mice
Source: Front Microbiol. 2026 Jan 6;16:1643268. doi: 10.3389/fmicb.2025.1643268 (PMC12815767; doi:10.3389/fmicb.2025.1643268)
Supplement: Supplementary file 2 [file Table_2.pdf]

Health	control	JFAT-L	JFAT-M	JFAT-H	cluster	Metabolites
0.62082354	-0.270996446	0.019694686	1.132903332	-1.502425112	7	Citraconic acid
-0.421494686	-0.685370385	1.768786613	-0.380627256	-0.281294286	6	Glyceraldehyde
-0.228444372	0.317853043	-1.56888913	1.121144409	0.35833605	4	Methylsuccinic acid
-0.192539716	0.611504303	-1.671249776	0.549311505	0.702973684	4	Oleic acid
1.253561923	0.759112294	-1.248077791	-0.348173378	-0.416423048	3	Uracil
-0.601217535	0.3991304	-1.448321002	0.726019478	0.924388659	4	Isopalmitic acid
0.245396231	0.770094662	-1.654998586	0.768769179	-0.129261487	2	Eicosadienoic acid
-0.698509772	-0.369348386	-1.053100614	1.153785094	0.967173678	5	16-Methylheptadecanoic acid
0.933296436	0.178631491	-1.514212301	0.802512831	-0.400228456	2	11Z-Eicosenoic acid
-0.816587144	0.644688539	-1.250448552	0.297407446	1.124939711	4	Palmitoleic acid
-0.168775262	0.855934549	-1.538242509	0.930778989	-0.079695768	4	10E,12Z-Octadecadienoic acid
-0.607426361	0.857755994	-1.459975756	0.731584238	0.478061885	4	Alpha-Linolenic acid
-0.886550278	0.90003777	-1.224470907	0.312753599	0.898229816	4	Myristoleic acid
1.653948534	-1.031778273	-0.078986197	-0.119729331	-0.423454733	8	L-Norleucine
1.264327238	0.784994642	-1.088061233	-0.216649469	-0.744611179	3	L-Lactic acid
1.089444886	-1.156234351	-0.115925788	0.941681314	-0.758966061	8	Urocanic acid
-1.591716045	0.204672712	1.127032561	0.375508866	-0.115498095	6	Hydrogen phosphate
0.310848863	1.308733382	-1.466290488	-0.196714489	0.043422732	9	9-Decenoic acid
1.460810445	0.598333591	-0.790725252	-0.436294983	-0.832123801	3	Threonic acid
-0.807661221	0.282089346	-1.245391815	0.612578945	1.158384745	4	Ethyl dodecanoate
1.552338682	-0.799571311	0.264478235	-0.082250145	-0.934995461	8	D-Alanine
0.245447901	-0.234925731	-0.993798862	-0.606886238	1.59016293	1	Xanthine
0.226647324	-1.174851402	1.525900725	-0.097647588	-0.480049059	6	o-Cresol
-1.45386226	0.429634438	0.071462854	-0.311878671	1.264643639	5	Eicosapentaenoic acid
-0.001664661	0.146243257	-1.528475757	0.106804871	1.27709229	1	16-Hydroxy hexadecanoic acid
0.992724633	0.398712159	-1.529249919	0.57260532	-0.434792192	2	9,10-Epoxyoctadecenoic acid
-0.257752685	-1.490298623	0.61363888	-0.021255813	1.155668241	5	Benzoic acid
0.386434289	-0.077464645	-1.542199255	0.022535016	1.210694594	1	2-Hydroxystearic acid
1.400542834	-1.30162555	0.401936278	-0.081206837	-0.419646725	8	D-Proline
-0.497807293	-0.463368862	1.78769892	-0.417723859	-0.408798906	6	3-Hydroxybutyric acid
0.409940359	0.806546652	-1.610322681	0.702258807	-0.308423137	2	Linoleic acid
0.645019071	1.241664196	0.026242712	-1.281954036	-0.630971942	3	Taurocholic acid
-0.062656385	0.231108422	-1.467650756	1.336878662	-0.037679943	2	2-Ketobutyric acid
0.496728935	0.014079158	-0.19244252	-1.512946004	1.194580432	1	Indoxyl sulfate
-1.14082563	-0.706647457	-0.090676115	1.364506599	0.573642603	5	(2E)-Decenoyl-ACP
0.512204364	1.191559845	-1.378457038	0.264415286	-0.589722457	9	Tetradecanedioic acid
1.516555122	-0.615108757	-0.704110201	0.536335785	-0.733671949	8	Thymidine
0.83673779	0.580194921	-1.654386	0.4334159	-0.195962611	2	Gamma-Linolenic acid
1.248474891	0.576235132	-1.313374089	-0.611743662	0.100407728	3	Xanthosine
1.36480944	-1.115189172	-0.778267826	-0.007901655	0.536549214	8	Dihydrojasmonic acid
0.79291491	0.850858264	-1.62641196	-0.03998229	0.022621076	9	Orotidine
0.168242403	0.440803595	-1.763701184	0.569249207	0.585405979	4	Succinic acid semialdehyde
0.321483006	0.843598663	-1.737493532	0.252452934	0.319958928	9	Uridine
0.05529287	0.003480903	-0.52490336	1.577082771	-1.110953184	7	15-Methylpalmitate
0.846435487	-1.189317932	-0.577522451	1.205844807	-0.285439911	7	Prostaglandin B2
0.380014661	1.116513587	-1.612627021	0.029058908	0.087039866	9	Docosahexaenoic acid
-0.970965645	0.57729631	-1.203500826	0.804250127	0.792920034	4	Allantoin
0.529670795	1.50149633	-0.907328557	-0.490484182	-0.633354385	9	Undecylenic acid
-0.731250208	-0.584411746	-0.597223121	1.641094249	0.271790826	5	Kynurenic acid
-0.449115288	0.05239528	-1.446675792	0.87912263	0.964273171	4	2-Oxovaleric acid
1.216445057	-0.380342718	-1.467314299	0.423715459	0.207496499	2	Hexadecanedioic acid
1.322009777	0.546199474	-1.099989812	-0.857870283	0.089650844	3	D-Glutamine
0.857340417	0.597256594	-1.359854761	0.678768996	-0.773511246	2	L-Glutamic acid
0.568912286	-0.060518691	-1.461904125	1.211915608	-0.258405079	2	(9xi,10xi,12xi)-9,10-Dihydroxy-12-octadecenoic acid
0.061911133	-0.129840852	-1.107330121	1.603137311	-0.427877471	2	Nervonic acid
-0.813330145	-0.490414002	1.733495552	-0.197012104	-0.232739301	6	Terephthalic acid
1.337296606	0.74951515	-0.605095949	-0.435912947	-1.04580286	3	5-Methylcytidine
0.299711536	0.940819227	-1.674804737	0.467900152	-0.033626177	2	7-Methylguanosine
1.205763992	0.946226058	-0.679002978	-0.507475044	-0.965512028	3	13,14-Dihydro-15-keto PGF2a
-0.537306096	-0.514932621	1.782416134	-0.400864041	-0.329313377	6	Pelargonic acid
-0.426699522	-0.531484755	-0.382289113	1.786227746	-0.445754356	7	N-Acetylserine
-0.550939529	0.306150357	-1.423118365	1.139333808	0.52857373	4	D-Xylose
-0.197054022	-0.40184349	1.752423399	-0.754797723	-0.398728164	6	2-Furoic acid
0.113256236	-0.567313413	-0.499723944	1.693598266	-0.739817146	7	2-Hydroxybutyric acid
-0.070465038	0.14217421	-1.412965316	-0.063833011	1.405089155	1	Inosine
1.011059811	0.909344668	-0.935903278	0.136364576	-1.120865777	3	Thromboxane B2
-0.441427424	1.544207891	-1.189168447	0.080009631	0.00637835	9	Succinic acid
-0.069804512	-1.14551538	-0.721390207	0.589478415	1.347231684	5	Hippuric acid
0.602799893	1.452329875	-0.608716824	-0.487856892	-0.958556052	3	Prostaglandin F2a
1.067829874	-0.011618188	-1.414121257	0.809754207	-0.451844636	2	9,10-DHOME

0.307151526	1.574270356	-0.276474184	-0.979905888	-0.62504181	9	Phenol glucuronide
1.752272355	-0.591565457	-0.443141056	-0.609549286	-0.108016556	8	2-Indolecarboxylic acid
0.033383745	1.346289637	-1.225220881	-0.657439895	0.502987394	9	Prostaglandin G2
1.347002104	-0.767428176	-0.443679648	0.765885598	-0.901779878	8	Indolelactic acid
1.577971883	0.083589104	-0.933001744	0.064162942	-0.792722185	3	myo-Inositol
1.480652018	-1.162385372	-0.161061621	0.378689677	-0.535894701	8	L-Phenylalanine
1.207884047	0.714368921	-1.243558847	0.01698264	-0.695676762	3	12-HETE
0.368896276	-0.245062951	-1.303436992	1.429200216	-0.249596548	2	Carnosol
-0.776696538	-0.408571049	1.750846914	-0.329732613	-0.235846714	6	Phthalic acid
0.375945924	1.321688622	-1.368954759	0.138770248	-0.467450034	9	5-KETE
1.40211377	-0.693616008	0.607810359	-0.25990361	-1.056404511	8	Pantothenic acid
0.42458446	0.727112761	-1.185766131	0.98770778	-0.95363887	2	Prostaglandin F3a
1.719707445	-0.628344936	-0.556577097	0.044867731	-0.579653143	8	Deoxyuridine
1.566687632	-0.99773547	-0.711593996	-0.058051483	0.200693316	8	p-Chlorobenzenesulfonamide
-0.560508827	1.747135905	-0.717866514	-0.298198069	-0.170562495	9	Pseudouridine
-0.50797871	-0.467069428	1.787139978	-0.396353168	-0.415738673	6	Citramalic acid
0.287677168	0.591306619	-1.578095653	0.994859636	-0.295747771	2	(10E,12Z)-9-HODE
-0.620763871	-0.550124852	-0.919346407	0.669434456	1.420800674	5	Cinnamoylglycine
0.981324675	-1.641825031	0.540062205	0.205980163	-0.085542013	8	N-Acetylornithine
-1.182094115	-0.361770029	-0.106107534	1.566374357	0.083597321	5	Rhamnose
-0.289472745	1.706917947	-0.942189386	-0.269106231	-0.206149585	9	Hexylresorcinol
-0.233211777	0.534159137	-1.590232938	1.032145587	0.257139991	4	D-Ribose
0.08657253	0.585471357	-1.679824551	0.903957221	0.103823442	2	(R)-3-Hydroxy-tetradecanoic acid
-0.851918644	1.644538011	-0.683294885	-0.274732155	0.165407671	9	12-Hydroxydodecanoic acid
0.137424537	0.778160445	-1.522137421	-0.358057872	0.964610311	1	trans-Aconitic acid
1.262690528	-1.358888824	0.64051787	-0.254429123	-0.289890452	8	2-Hydroxycinnamic acid
-0.581610751	-0.519907069	1.778195738	-0.354134347	-0.322543571	6	Nicotinic acid
1.771459905	-0.611616312	-0.521493546	-0.397131366	-0.241218682	8	L-Arginine
-0.588946654	-0.625112925	1.763473976	-0.246360088	-0.303054308	6	3-(3,4-Dihydroxy-5-methoxy)-2-propenoic acid
-0.781820663	0.993403782	-0.816905834	1.183389023	-0.578066308	4	L-Fucose
0.558128481	-1.137618039	-0.759957095	1.347854708	-0.008408055	7	(R)-mandelic Acid
0.43391488	-1.627754619	0.021468308	1.073240386	0.099131044	7	Itaconic acid
-0.347640031	0.303560445	0.352667944	1.190140535	-1.498728893	7	3-Furoic acid
-0.02397675	-1.066870977	0.482791651	1.409301208	-0.801245131	7	D-Xylulose
0.682662222	-0.113358264	-1.466241718	1.144532364	-0.247594604	2	N-Acetyl-L-alanine
1.037691263	-1.604067206	-0.014988523	0.591471415	-0.01010695	8	4-Hydroxycinnamic acid
-0.124291491	0.123453671	-0.939160726	1.620105641	-0.680107094	2	Erucic acid
1.421758663	0.357798897	-0.918500538	0.133488076	-0.994545097	3	8,15-DiHETE
-0.327018644	1.680302906	-1.003895764	-0.194670305	-0.154718193	9	3-Hydroxycapric acid
-0.459934151	0.177342888	-1.46313743	1.0878697	0.657858992	4	Deoxyribose 5-phosphate
0.031809703	1.281329259	-1.400334906	-0.399371584	0.486567529	9	Jasmonic acid
0.198372678	-0.646006452	-0.349588739	1.644269675	-0.847047162	7	Ribitol
0.146249679	0.58987305	-1.486195544	1.130211555	-0.380138741	2	N-Acetyl-L-aspartic acid
0.406984533	1.487391403	-1.041283208	-0.72139894	-0.131693789	9	Perillic acid
1.392686346	0.588676838	-0.642860834	-0.219351186	-1.119151164	3	Hypotaurine
1.014058087	-1.638539901	-0.011274153	0.523612348	0.112143619	8	Phenylpyruvic acid
-0.722693155	-0.427533599	1.724897067	-0.009363261	-0.565307052	6	Ricinoleic acid
-0.350135104	1.748253417	-0.790382829	-0.227581524	-0.38015396	9	Acetylglycine
-0.180196902	1.47423264	-1.338864597	0.005036616	0.039792243	9	Hydroxyisocaproic acid
1.214194806	-0.748563503	-0.880890284	-0.535066931	0.950325913	1	Indole-3-propionic acid
0.391800908	-1.298111518	1.210020779	0.418727213	-0.722437382	6	4-Hydroxyphenylpyruvic acid
-0.822147897	0.434354057	-0.982386927	1.469828444	-0.099647676	4	Calcidiol
1.686857748	-0.100114187	-0.410312886	-0.965134996	-0.211295678	3	L-Lysine
-1.420436959	0.807719363	0.748246398	0.549038475	-0.684567277	6	2-(3,4-dihydroxyphenyl)-3,5,7-trihydroxy-3,4-dihydro-2H-1-benzopyran-4-one
0.621115833	-0.479100551	-1.048059456	-0.515427998	1.421472173	1	Sinapyl alcohol
0.078802109	-1.208381495	-0.272279781	-0.158390947	1.560250114	5	Benzyl acetate
0.647067325	0.112049009	-1.32608617	1.191647064	-0.624677228	2	2-acetyl-1-alkyl-sn-glycero-3-phosphocholine
0.475518149	0.813288109	-1.146976518	-1.016121718	0.874291978	1	Mesaconic acid
-0.526922887	1.622394678	-1.039516303	-0.091334381	0.035378893	9	(1R,2S,3R)-2-Acetyl-4(5)-(1,2,3,4-tetrahydroxybutyl)imidazole
-0.584789442	-0.125197394	1.734135178	-0.27682729	-0.747321053	6	Dehydroascorbic acid
0.017801058	0.372941125	-1.493924163	-0.162651054	1.265833034	1	Deoxyinosine
-0.334593356	1.645649137	-1.069167294	-0.182232429	-0.059656058	9	Methyl jasmonate
1.053445999	-1.274373948	0.884425739	0.031499659	-0.694997448	8	N-Methylalanine
1.613619375	-0.28145625	-0.897596491	0.239286147	-0.673852781	3	4-hydroxy-3-[1-(4-hydroxyphenyl)-3-oxobutyl]-2H-chromen-2-one
-0.621143269	1.11916131	-1.393779973	0.542331135	0.353430797	4	Dihydrolipoate
0.123695525	-1.227679797	-0.692169628	1.337006626	0.459147274	5	PE(16:0/18:2(9Z,12Z))

1.345732405	-1.448655592	0.202598719	0.099137774	-0.198813307	8	Protocatechuic acid
0.405904273	0.39914213	-1.598525449	1.031498994	-0.238019949	2	3-Methoxy-4-hydroxyphenylethyleneglycol
0.359988922	-0.199238191	-1.544947296	1.184972458	0.199224107	2	Ascorbic acid
1.63285699	-0.929848745	0.177910108	-0.283139782	-0.597778571	8	L-Allothreonine
0.777149805	1.071578173	-1.475449838	-0.210491636	-0.162786504	9	Cytidine
0.703742457	-1.446386474	1.110992313	0.050951954	-0.41930025	8	Mulberrin
0.555361617	0.452630177	-1.781740577	0.466737786	0.307010997	2	Sedoheptulose
-0.814384467	-1.252564059	0.227977929	0.807724721	1.031245876	5	1H-Indole-2,3-dione
1.428129446	-0.048296981	-0.940635951	0.479283315	-0.91847983	3	15-Keto-prostaglandin E2
1.082325868	1.032229258	-0.995008763	-0.289274563	-0.8302718	3	Dihydroxyacetone (dimer)
-0.860865784	0.372624503	-1.249615739	0.713287774	1.024569245	4	3-Sulfinato-L-alaninate
1.727504362	-0.562198462	-0.336619231	-0.065783249	-0.76290342	8	(Â±)-Tryptophan
0.333991721	0.955399251	-1.697492811	0.130205127	0.277896713	9	Pyrrole-2-carboxylic acid
1.451989413	-0.452736593	-0.282117683	0.463023733	-1.18015887	8	Orotidylic acid
-0.276769198	0.222682481	-1.46705804	0.229247927	1.29189683	1	3alpha-Acetoxy-11-keto-beta-boswellic acid
-0.517769138	-1.309862213	-0.135913997	1.168786472	0.794758875	5	5-Hydroxy-L-tryptophan
0.405670603	-0.487228994	-1.439992691	1.189087034	0.332464049	2	Protoporphyrin IX
0.082980229	-1.025034774	0.893223585	1.059639463	-1.010808503	7	1,5-Anhydrosorbitol
-0.429742032	-1.364419325	1.362453685	0.261766204	0.169941468	6	Dodecanedioic acid
0.802162871	-1.111272627	1.282060466	-0.534497733	-0.438452977	8	Pyridoxal
-0.851041626	-0.896997047	-0.312446223	0.67619245	1.384292446	5	Phenylglyoxylic acid
0.552651023	-0.288841313	-1.209378899	-0.449576418	1.395145607	1	12-Oxo-2,3-dinor-10,15-phytodienoic acid
0.78927658	0.506587262	-0.836080791	0.846138332	-1.305921384	2	L-Iditol
0.109842875	1.330731877	-1.40789109	-0.358683606	0.325999944	9	S-Nitrosoglutathione
